# Bioinformatic Analysis of Autism-Related miRNAs and Their PoTential as Biomarkers for Autism Epigenetic Inheritance

**DOI:** 10.3390/genes16040418

**Published:** 2025-03-31

**Authors:** Larissa Naísa Acerbi da Silva, Taiza Stumpp

**Affiliations:** Laboratory of Developmental Biology, Department of Morphology and Genetics–Paulista Medicine School, Federal University of São Paulo (UNIFESP), Sao Paulo 04021-001, Brazil; larissa.naisa@unifesp.br

**Keywords:** microRNA, autism, epigenetic inheritance

## Abstract

Background/Objectives: The dysregulation of miRNA expression in samples from autistic individuals indicates that they are involved in autism. The participation of miRNAs in paternal epigenetic inheritance has also been reported. This study used bioinformatics tools to analyze the literature and genetic databases to search for miRNAs associated with autism, aiming to explore their suitability to investigate paternal epigenetic inheritance. Methods: Autism-related miRNAs were searched in public databases using bioinformatic tools (miRNA-to-genes analysis). The genes targeted by these autism-related miRNAs, which are common to neurons, sperm, and PBMCs, were identified. Enrichment analyses were performed to identify the biological processes regulated by the candidate miRNAs. Autism-related miRNAs were also identified by an inverse analysis (genes-to-miRNA analysis), starting from autism-related genes. Results: In the miRNA-to-gene analysis, 416 miRNAs involved in autism were found, of which 77 were expressed in sperm, PBMCs, and neurons. From these, 18 were differentially expressed in the brain and in at least one peripheral sample (saliva or blood), suggesting that they might be suitable to be used in the investigation of autism biomarkers and inheritance. In the genes-to-miRNA analysis, 36 miRNAs were identified, from which 9 coincided with the results of direct analysis. Conclusions: Although there is no consensus about miRNAs related to autism, there are candidate miRNAs that show clear potential to be explored as biomarkers. The coincidence in the expression of miRNAs in sperm, neurons, and PBMCs indicates that they are valuable biological samples to study the role of miRNAs in the paternal epigenetic inheritance of autism.

## 1. Introduction

Autism, referred to in diagnostic manuals as autism spectrum disorder (ASD), is a neurodevelopmental condition characterized by difficulties in social interaction and social communication, as well as by restricted and repetitive patterns of behaviors, interests, or activities [1]. Autism manifestation varies greatly between individuals, as does the level of support required, and may also be accompanied by symptoms typical of other conditions, including intellectual disability, attention deficit hyperactivity disorder (ADHD), and anxiety disorder. Other characteristics may also be present, including language impairment, epilepsy, gastrointestinal problems, motor deficits, and sleep disorders [1,2,3,4,5,6], leading to great complexity.

Autism is a multifactorial condition that involves genetic factors, environmental influences, and epigenetic mechanisms [7]. The epigenetic mechanisms include DNA methylation, histone modification, and the activity of small non-coding RNAs. MiRNAs, the focus of this study, are sncRNAs with approximately 22 nucleotides that regulate gene expression through interaction with mRNA [8]. During neurodevelopment, interactions between miRNAs and their target genes regulate important processes, such as neurogenesis, gliogenesis, neuronal migration, neuronal polarization, and dendrite and synapse formation [9]. Dysregulated patterns of miRNA expression in autistic individuals have already been suggested by studies using different types of samples, including post-mortem brain, saliva, blood plasma and serum, peripheral blood monocytes, olfactory mucosa stem cells, lymphoblastoid cell lines, and neurons derived from induced pluripotent stem cells [10,11]. Ozkul and colleagues [12] analyzed the expression of miRNAs in serum samples obtained from 45 autistic children and found 6 miRNAs with low or very low expression compared to the control participants.

One of the methods used to study cellular and molecular mechanisms involved in autism and possible biomarker exploration is the collection of peripheral mononuclear blood cells (PBMCs) and its reprogramming into induced pluripotent stem cells [13,14,15,16]. In addition, PBMCs are considered a good peripheral sample to investigate systemic alterations related to autism [17,18,19,20,21,22], including miRNA dysregulation [23,24,25,26,27,28,29].

Despite the large number of studies seeking to comprehend autism etiology, this condition remains obscure, although its high heritability seems clear. Large-scale genetic studies indicate that both rare and common variants are related to the etiology of autism, although common genetic variants are responsible for most cases and are more frequently involved in the heritability of autism [30]. The participation of miRNAs in epigenetic inheritance has also been reported. Animal studies have suggested that miRNAs present in sperm can contribute to paternal epigenetic inheritance of behaviors across generations [31,32,33,34]. Regarding autism paternal inheritance, Ozkul and colleagues [12] compared the expression of the six miRNAs between sperm samples from the father of one of the autistic participants and samples from the control men; they observed that five of them showed reduced expression. Dysregulation of the miRNA expression in sperm can be caused by different environmental factors, such as smoking [35] and early life stress [36], and may have repercussions for the descendants. However, the mechanisms through which this type of inheritance occurs and how environmental factors can influence autism and are transmitted to subsequent generations are still poorly understood.

Although data regarding the participation of miRNAs in autism etiology and inheritance is promising and bulk data have been produced, it is still controversial, with studies reporting conflicting information about the role of specific miRNAs. Considering that the studies about the epigenetic inheritance of autism involve a comparison of paternal and child biological samples, and that one of the most used samples are PBMCs, it is important to know the miRNA profile of these samples and how they reflect miRNA signatures of the autistic brain. To address this issue, this study used bioinformatics tools to analyze the literature and different genetic databases to search for miRNAs associated with autism, as well as to explore their expression in PBMCs and sperm, aiming to infer the suitability of these cell types to investigate paternal epigenetic inheritance via miRNAs.

## 2. Materials and Methods

**Figure 1:**
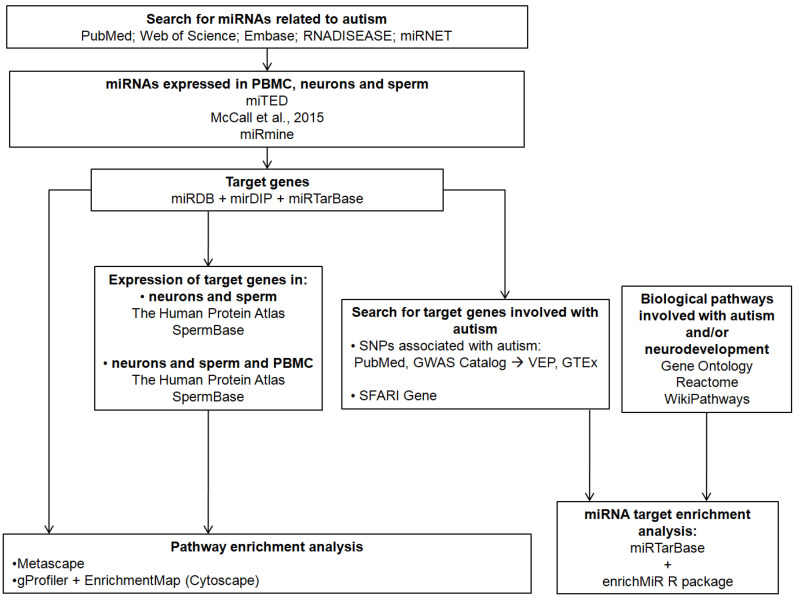
Workflow of the methods used in the current study [37,38,39,40,41,42,43,44,45,46,47,48,49,50,51,52,53,54].

Figure 1 shows a workflow of the methods used in the current study.

### 2.1. Compilation of Autism-Related miRNAs

Autism-related miRNAs were obtained from miRNet version 2.0 [37] and RNADisease version 4.0 [38], and the PubMed, Web of Science, and Embase databases. Medical Subjective Heading terms related to autism and general terms referring to autism, such as “Autism spectrum disorder”, “Autistic disorder”, and “autism”, were used in combination with the terms “miRNA”, “microRNA”, and “sncRNA” (Table S1). The searches were carried out from December 2022 to February 2023. Non-experimental studies that analyzed previously published databases were also included.

The following data were extracted from the articles: DOI, PMID, name of the authors, year of publication, type of biological sample used, statistical test used, characteristics of the participants (number of volunteers in the ASD and control groups; age; inclusion and exclusion criteria), method used to identify miRNAs (RNA sequencing, microarray, PCR, NanoString nCounter, TaqMan low-density array), and differentially expressed miRNAs in samples of autistic individuals compared to the control participants or those suggested as potential biomarkers for this disorder.

The names of the mature miRNAs that resulted from these searches were manually standardized according to the classification adopted in miRBase [55] version 22.1; the miRBase Converter package version 1.30 [56] in R (version 4.2.2) and RStudio (version 2023.12.0+369) was also used to convert the names of the miRNAs. The miRNAs absent in miRBase were not included in the subsequent analyses.

### 2.2. Identification of miRNAs Expressed in Peripheral Blood Mononuclear Cells (PBMCs), Sperm, and Neurons

One desirable characteristic for the identification of suitable biomarkers for autism and its paternal inheritance is the coincidence of these miRNAs in peripheral samples, in the central nervous system, and in sperm. The peripheral samples chosen for this analysis were peripheral blood mononuclear cells (PBMCs), since they are naturally obtained when blood is collected to perform general laboratorial and/or genetic analyses of autistic individuals. The identification of miRNAs expressed in each of these three cell types was performed using miTED [39] for PBMC analysis, miRmine [40] and SpermBase [41] for sperm analysis, and data provided by McCall and collaborators [42] for neuron analysis. For all three cell types, only miRNAs with an expression ≥100 RPM were considered expressed. All miRNAs available in SpermBase were considered as expressed in sperm.

In addition to the coincident expression in these three cell types, another criterion used to identify possibly relevant miRNA was its expression in the post-mortem brain and in one type of peripheral sample (blood, plasma, serum, PBMCs, or saliva) of autistic individuals.

The expression of the miRNA provided by the databases mentioned above was compared between the three cell types to identify miRNAs whose expression coincides between them. This analysis was performed using the R program (version 4.3.2) and RStudio [57], as well as for the intersection of the sets of miRNAs. The packages used were: ”readxl” [58], “dplyr” [59], “tidyr” [60], “DescTools” [61], and “ggvenn” [62].

### 2.3. Analysis of Genes Targeted by the Autism-Related miRNAs

To confirm whether the compiled miRNAs target genes were relevant to autism, two tools were used: miRDB [43], mirDIP version 5.3.0.1 [44], and miRTarBase version 9.0 [45]. A default score threshold of 50 was adopted to select miRDB target genes. For targets predicted by mirDIP, the minimum score class “high” was selected.

After retrieving the target genes, the gene symbols were converted to Ensembl ID using R (version 4.3.2), RStudio, and the package “gprofiler2” (version 0.2.2) [63]. The conversion of the gene symbols to the Ensembl ID was carried out due to the existence of distinct symbols to represent the same gene and the existence of different genes with coincident symbols.

For subsequent analysis, the target genes were separated into three groups:

(a) all genes retrieved;

(b) genes expressed in PBMCs, neurons, and sperm;

(c) genes expressed only in neurons and sperm.

### 2.4. Pathway Enrichment Analysis

#### 2.4.1. Metascape

To analyze the biological pathways and diseases in which the genes targeted by the autism-related miRNAs are involved, the Metascape portal, release 3.5, was used [46]. Metascape uses a hypergeometric test for the enrichment analysis. This tool clusters enriched terms into non-redundant groups using the Kappa-test score, and the term with the lowest *p*-value within each cluster is chosen to represent the cluster. Process enrichment was performed using Gene Ontology (Biological Process), Wikipathways, KEGG, and Reactome ontologies. Disease enrichment was also performed using the DisGeNET database. Three different sets of gene lists were used for enrichment analyses using these tools, as follows:

(a) all the genes targeted by each miRNA;

(b) target genes expressed in common in sperm and neurons;

(c) target genes expressed in common in PBMCs, sperm, and neurons.

Thus, a previous analysis of the genes that are specifically expressed in PBMCs, neurons, and sperm was necessary. For this, RNA expression data available in the Human Protein Atlas [47] were obtained for total PBMCs, for inhibitory neurons, and excitatory neurons. Gene expression data in sperm were obtained from SpermBase. For Human Protein Atlas data, genes with an expression ≥1 nTPM (normalized transcripts per million) in inhibitory or excitatory neurons and in PBMCs were considered as expressed; and for SpermBase data, all coding genes were considered as expressed in sperm. The gene symbols in the SpermBase data table were converted to Ensembl ID using R (version 4.3.2), RStudio [57], and the package “gprofiler2” (version 0.2.2) [63].

For the enrichment analysis (a), *p*-value = 0.01, minimum number of genes in the term = 3, enrichment score = 1.5, and Kappa score = 0.3; standard values defined by Metascape release 3.5 were adopted. The default *p*-value of 0.01 was adopted because it is a *p*-value rigorous enough to provide coherent, legitimate, and enriched terms. For the other analyses from (b) to (c), *p*-value = 10^−4^, enrichment score = 1.5, minimum number of genes in the term = 3, and Kappa score = 0.3 were adopted. A stricter *p*-value was chosen to avoid interpreting enrichment analysis results with marginal *p*-values, such as 10^−3^ or 10^−4^ [64].

Within Metascape release 3.5, the “multi-gene list meta-analysis” tool was used to compare process enrichment between the lists of analyses (b) and (c); however, it was not used for analysis (a), since performing enrichment analysis for target genes of all miRNAs would exceed the limit imposed by Metascape release 3.5 (3000 genes).

For the analysis (a), the entire genome was used as the background. To avoid results that included processes unrelated to the cells of interest, restricted background lists were adopted in the analyses considering these specific cell types. Therefore, for analysis (b), only genes expressed in common in neurons and sperm were considered for the enrichment background, and for analysis (c), only genes expressed in common in PBMCs, neurons, and sperm were considered as background.

#### 2.4.2. gProfiler

In addition to Metascape release 3.5, enrichment analysis was also performed using gProfiler [48], including the databases Gene Ontology (Biological Process), Reactome, WikiPathways, and Human Phenotype Ontology. As pathways with few genes can be difficult to interpret results and present redundancy with larger pathways, terms with fewer than 10 genes were excluded. Furthermore, terms from the biological pathway databases (Gene Ontology, Reactome, and WikiPathways) with more than 500 genes and Human Phenotype Ontology terms with more than 1000 genes were also excluded, since terms containing many genes are overly general and do not contribute significantly to the interpretation of results.

As performed for Metascape, the three gene groups (a, b, and c) previously described were analyzed in gProfiler. To correct the *p*-value in gProfiler, gSCS was used, and a corrected *p*-value less than 0.05 was adopted. For analysis (a), only annotated genes were included as the background, and for the other analyses, the customized backgrounds described in the previous section were used, although they were customized over annotated genes.

To visualize the results of this enrichment analysis, enrichment maps were created using the EnrichmentMap library [49] in Cytoscape software, version 3.10.1 [50]. Maps for biological pathways and maps for phenotypes were performed separately. The Gene Matrix Transposed file was obtained from gProfiler on 22 February 2024. The creation of the enrichment maps was based on the “Pathway enrichment analysis and visualization of omics data using g:Profiler, Gene Set Enrichment Analysis (GSEA), Cytoscape, and EnrichmentMap” protocol [65].

The clustermaker2 app, version 2.3.4, was applied to define clusters using the Markov Clustering Algorithm, and the number of clusters was limited to a maximum of 20. For analysis (a), i.e., for all genes targeted by all candidate miRNAs (described in the previous section), the clusters were defined separately for each miRNA to identify relevant biological pathways regulated by each one individually, and clusters were also defined using the results of all enrichment analyses to compare the miRNA target gene lists. In the clusters defined individually for each miRNA, the term with the lowest adjusted *p*-value was selected to represent each cluster. In other analyses, the AutoAnnotate app, which uses the WordCloud app to name the clusters, was used, and, when necessary, the name of the cluster was changed to better describe the terms included. The parameters adopted in the creation of the enrichment maps are shown in Table 1.

### 2.5. miRNAs Enrichment Analysis

To validate autism-related miRNAs that resulted from the active search, an inverse analysis was carried out, starting with the genes suggested to be associated with autism to then find the miRNAs that target them. Autism-related genes were searched in SFARI [51], in the literature using PubMed, and in the GWAS Catalog [52]. Although GWAS can consider different types of genetic variations, the single nucleotide polymorphisms (SNPs) are the most studied [66]; thus, only GWAS and GWAS meta-analyses that analyzed SNPs were considered. In addition, only associations with a *p*-value < 5 × 10^−8^ were included. The searches were carried out from April 2023 to June 2023. From the list of SNPs found, two criteria were adopted to identify the genes influenced by these autism-related SNPs: the proximity of the SNP and expression quantitative trait loci (eQTL). For proximity analysis, the Variant Effect Predictor [53] was used. In the Variant Effect Predictor tool, genes that contained at least one of the variants in intron regions, exon regions, the 3′ UTR region, or with variants up to 5000 base pairs away from the gene were included. For eQTL analysis, the GTEx (version 8—https://gtexportal.org/home/ (accessed on 29 April 2024)) was accessed to determine whether any of the significant genome variants corresponded to eQTL. Data from all regions of the brain with eQTL data available in GTEX were used: amygdala, anterior cingulate cortex (BA24), caudate nucleus (basal ganglia), cerebellum, cerebellar hemisphere, frontal cortex (BA9), cortex, hippocampus, hypothalamus, nucleus accumbens (basal ganglia), putamen (basal ganglia), and substantia nigra. GTEx and Variant Effect Predictor were accessed on 29 April 2024.

Genes available in the SFARI Gene database were downloaded on 2 May 2024. The symbols of the genes from SFARI Gene were converted to Ensembl ID using R (version 4.3.2), RStudio [57], and the “gprofiler2” package(version 0.2.2) [63].

Neurodevelopment-related genes were also obtained from the Gene Ontology (https://geneontology.org/ (accessed on 9 April 2024)), WikiPathways (https://www.wikipathways.org/ (accessed on 9 April 2024)), or Reactome version 88 (https://reactome.org/ (accessed on 9 April 2024)) portals, considering 11 biological pathways (8 biological processes from Gene Ontology, 2 biological pathways from WikiPathways, and 1 biological pathway from Reactome—Tables S2 and S3). The downloads were performed on 9 April 2024. The biological pathways were selected according to their relationship with brain development, neurogenesis, and synapse function. Pathway gene symbols were converted to Ensembl ID using R (version 4.3.2), RStudio, and the “gprofiler2” package (version 0.2.2) [63].

Enrichment analysis was performed using the “enrichMiR” package version 0.99.32 [54] in R (version 4.3.2) and RStudio. All miRNA-gene interactions experimentally validated for humans and available in miRTarBase 9.0 were downloaded [45]. The background gene list included all human protein-coding genes (GRCh38.p14) from Ensembl 111 [67] and was obtained through BioMart. In the “enrichMiR” package, the overlap test (Fisher test) was performed; only genes present in common in the background and in the miRTarBase were considered (14,479 genes) and only miRNAs with at least five experimentally validated interactions were included (2602 miRNAs). An FDR = 0.05 was adopted. The R script used is available in Additional File S1.

## 3. Results

### 3.1. Autism-Related miRNAs: miRNA-to-Gene Analysis

A total of 749 miRNAs were identified in these studies. These miRNAs were identified in different types of samples, such as serum, plasma, PBMCs, monocytes, lymphoblastoid cells lines, post-mortem brain, cerebellar cortex, saliva, olfactory mucosal stem cells, fibroblasts, neural stem cells, neural progenitor cells, astrocytes, and neurons derived from induced pluripotent stem cells and pineal gland samples. From these 749 miRNAs, 56 had no record in miRBase and were, therefore, not included in the subsequent analyses. After the update according to miRBase and the exclusion of duplicates (the same miRNAs identified in more than one study), 416 different mature miRNAs were obtained (Table S4). The most cited miRNAs were hsa-miR-23a-3p, hsa-miR-451a, and hsa-miR-7-5p.

### 3.2. miRNAs Expressed in PBMCs, Neurons, and Sperm

**Figure 2:**
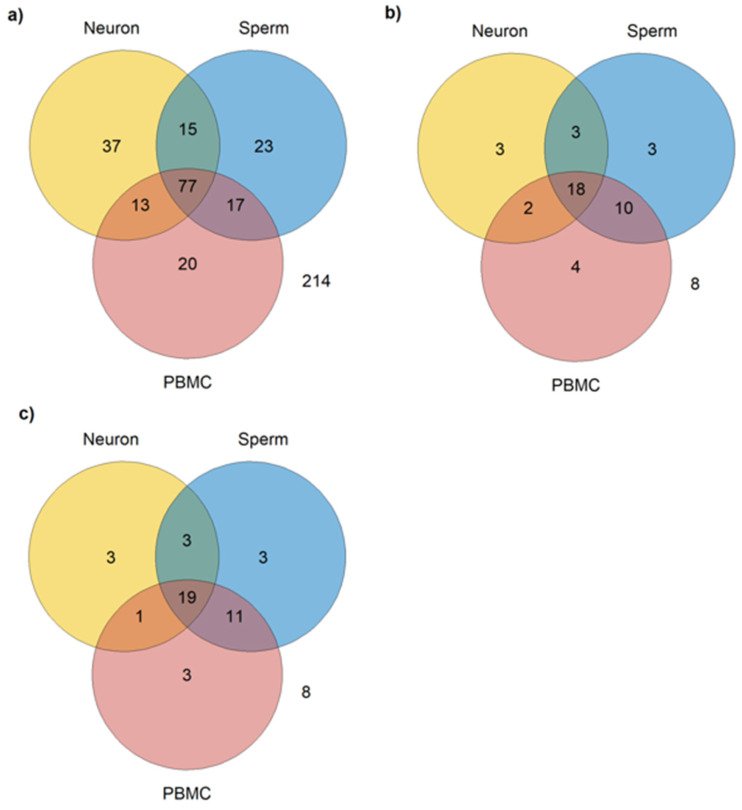
Number of miRNAs expressed in sperm, neurons, and PBMCs. (**a**) Total number of miRNAs expressed in common in sperm (miRmine), neurons, and PBMCs, regardless of the type of tissue used in the studies included; (**b**) number of miRNAs expressed in common in sperm (miRmine), neurons, and PBMCs reported as dysregulated in post-mortem brain or cerebellum and in peripheral samples (blood, plasma, serum, PBMCs, or saliva); (**c**) number of miRNAs expressed in common in sperm neurons and PBMCs in post-mortem brain or cerebellum samples and in peripheral samples (blood, plasma, serum, PBMCs, or saliva).

Considering the criteria adopted, from the 416 autism-related miRNAs identified, 77 were identified as common to PBMCs, neurons, and sperm (Table S5); 17 are expressed in PBMCs and sperm, but not in neurons; 15 are expressed in neurons and sperm, but not in PBMC; and 13 are expressed in PBMCs and neurons but not in sperm; and finally, 214 miRNAs were not identified as expressed in any of the three cell types (Figure 2a).

As described in the Methods section, in addition to expression in the aforementioned cell types, another criterion was adopted for the selection of the most relevant miRNAs as biomarkers for autism and its inheritance: its dysregulation in at least one study that used post-mortem brain or cerebellum and in one study that used viable peripheral samples (blood, plasma, serum, PBMCs, or saliva), i.e., that can be collected in a low or non-invasive way. A total of 51 miRNAs met these criteria, from which 3 are expressed only in neurons (Table 2, Figure 2b), 3 only in sperm (Table 2, Figure 2b), 4 only in PBMCs (Table 2, Figure 2b), 2 in neurons and PBMCs (Table 3, Figure 2b), 10 in PBMCs and sperm (Table 3, Figure 2b), 3 in neuron and sperm (Table 3, Figure 2b), and 8 are not expressed in any of the three cell types (hsa-miR-1277-3p, hsa-miR-33b-5p, hsa-miR-219a-5p, hsa-miR-940, hsa-miR-4742-3p, hsa-miR-4454, hsa-miR-206, and hsa-miR-1248).

According to SpermBase, from the 51 autism-related miRNAs found, 36 are expressed in sperm (Figure 2c). However, a difference in sperm-expressed miRNA was found between Spermbase and miRmine: has-miR-4454, has-miR196a-5p, has-miR-494-3p, has-miR-541a, has-miR-664a-3p, and has-miR-199a-5p were found in SpermBase, but not in miRmine. On the other hand, four miRNAs found in miRmine (hsa-miR-193b-3p, hsa-miR-379-5p, hsa-miR-148a-5p, and hsa-miR-142-5p) were not found in SpermBase. However, 30 miRNAs were found in both databases (Table 4).

When miRmine and Spermbase data were combined, 18 miRNAs were found expressed in common in the three cell types (Table 5, Figure 2b).

### 3.3. Identification of Genes Targeted by Candidate Autism-Related miRNA

From the 18 autism-related miRNAs expressed in the 3 cell types (neurons, PBMCs, and sperm; Table 5), the one with the highest number of target genes was hsa-miR-335-3p (2549 genes). Table 6 shows the total number of genes targeted by autism-related miRNAs predicted by miRDB or mirDIP, experimentally validated according to miRTarBase, and expressed in common in sperm/neurons or in sperm, PBMCs, and neurons.

Among the target genes that resulted from this analysis, there were genes regulated by more than one miRNA of interest. *TNRC6B*, for example, is targeted by 16 of the 18 miRNAs, representing the gene targeted by the largest number of autism-related miRNAs of interest. Other relevant genes targeted by multiple autism-related miRNAs include *KMT2A* (targeted by 13 miRNAs), *PTEN* (targeted by 13 miRNAs), *AGO1* (targeted by 12 miRNAs), *CLOCK* (targeted by 12 miRNAs), *MECP2* (targeted by 10 miRNAs), and *ABCA1* (targeted by 10 miRNAs). All the genes targeted by the 18 miRNAs are presented in Table S6.

### 3.4. Enrichment Analysis of All the Genes Targeted by the 18 Candidate Autism-Related miRNAs

To access the biological pathways related to the 18 miRNAs obtained from previous analysis, enrichment analyses were performed using the genes targeted by these miRNAs. For this, two distinct approaches were adopted: one using Metascape release 3.5 and another using gProfiler, followed by the creation of enrichment maps in Cytoscape version 3.10.1 and clusters using clustermaker2 version 2.3.4.

In Metascape analysis, pathways related to the nervous system or neurodevelopment appeared as relevant terms (Supplementary Figures S1–S18). The number of terms related to the nervous system or neurodevelopment in enrichment analysis are shown in Table 6. The representative terms related to the greater number of miRNAs compared to other term pathways were “head development” (GO:0060322), “enzyme-linked receptor protein signaling pathway” (GO:0007167), “phosphorylation” (GO:0016310), and “tube morphogenesis” (GO:0035239). Complete Metascape results are shown in the Table S7.

In the analysis by gProfiler, the cluster that contained the largest number of terms was “axon extension” (27 terms), which included terms involved with cell growth and axonogenesis (Supplementary Figure S19a). Except for hsa-miR-23a-3p, the target genes of all other miRNAs were associated with at least one term belonging to this cluster. All clusters are shown in Table S8.

Clusters were also created for target genes of each miRNA individually (Table S9), and the gene ontology “forebrain development” appeared as the representative term associated with the greatest number of miRNAs (8 miRNAs) compared to other terms, followed by “ameboidal-type cell migration” (GO:0001667), “axonogenesis” (GO:0007409), “regulation of amide metabolic process” (GO:0034248), “response to transforming growth factor β” (GO:0071559), and “Wnt signaling pathway” (GO:0016055). Supplementary Figures S1–S18 show the results of the enrichment analysis performed individually for each miRNA target gene list, and the number of terms related to the nervous system or neurodevelopment in the enrichment analysis are shown in Table 7.

When the results from gProfiler for the Human Phenotype Ontology obtained using the list of genes targeted by each miRNA were used in Cytoscape, 20 clusters were defined. The cluster containing the largest number of terms was “Palpebral fissures” (18 terms), which included terms involved with abnormalities in the palpebral fissure, ear position, and nose morphology, among others (Supplementary Figure S19b, Table S10).

Databases for gene-disease associations were also used to perform the enrichment analysis for the miRNAs. Metascape and gProfile allow this analysis via the embedded databases DisGeNET and Human Phenotype Ontology, respectively. In DisGeNET, the terms “Neurodevelopmental disorders” and “Developmental delay (disorders)” were associated with the highest number of autism-related miRNAs. Supplementary Figures S20–S37 show the results of the enrichment analysis performed individually for each miRNA. The analysis using Human Phenotype Ontology pointed out the terms “Autistic behavior”, “Disinhibition”, and “Hyperactivity” as the ones associated with the largest number of autism-related miRNAs.

### 3.5. Enrichment Analysis of Target Genes Common to Neurons and Sperm

The identification of miRNAs potentially useful as biomarkers of autism paternal inheritance can be helpful. It is, therefore, important to analyze possible similarities in miRNA signatures between sperm, the carrier of paternal hereditary information, and neurons, where autism-related miRNAs are primarily considered.

**Figure 3:**
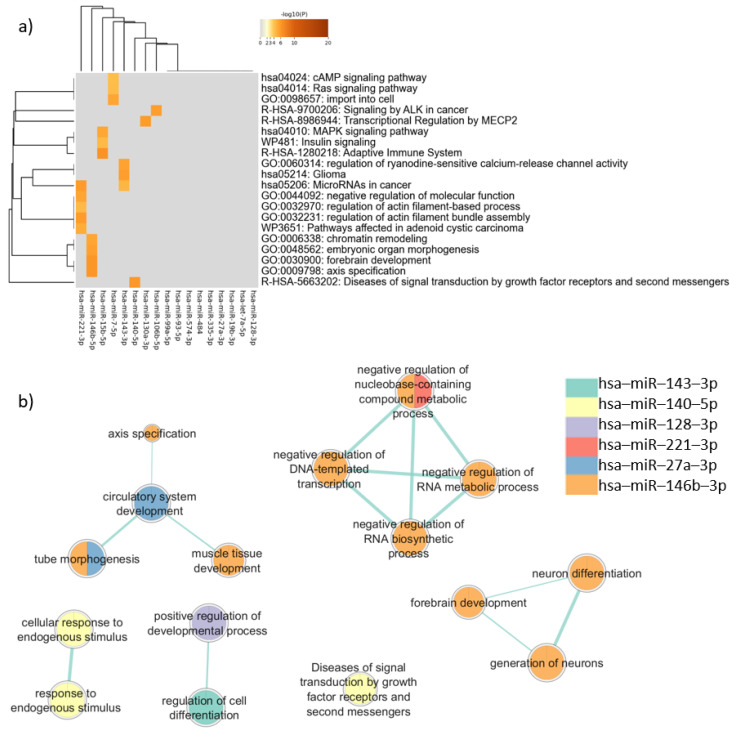
Pathway enrichment analysis for target genes expressed in common in sperm and neurons. (**a**) Heatmap of enriched terms across miRNAs target genes list, produced by Metascape; (**b**) Enrichment map of enriched terms for miRNAs target genes lists created using EnrichmentMap in Cytoscape and gProfiler results.

In the enrichment analysis performed for the target genes expressed in common in neurons and sperm using Metascape, “MAPK signaling pathway” (log(q) = −5.99) was the most representative term (Figure 3a), followed by terms related to cancer, such as “MicroRNAs in cancer” (log(q) = −4.80), “Pathways affected in adenoid cystic carcinoma” (log(q) = −3.69), “Glioma” (log(q) = −2.45), and “Signaling by ALK in cancer” (log(q) = −2.45). The term “forebrain development” (log(q) = −2.45) appeared for one miRNA (Figure 3). Clusters defined by Metascape are shown in Table B in Table S11.

In the enrichment analysis performed using gProfiler, only six miRNAs were associated with biological pathways. The genes targeted by hsa-miR-146b-5p presented more associations with relevant terms than genes targeted by the other miRNAs. Terms related to neurodevelopment were noted, such as “neuron differentiation” (padj = 0.011), “generation of neurons” (padj = 0.021), “forebrain development” (padj = 0.035), and “tube morphogenesis” (padj = 0.035) (Figure 3b).

Comparing the two different enrichment analysis tools, in addition to the genes targeted by has-miR-146b-5p, that had similar results between Metascape and gProfiler analysis, the analysis carried out by gProfiler showed that the target genes of hsa-miR-140-5p were associated with “Diseases of signal transduction by growth factor receptors and second messengers” (padj = 0.014), similar to the Metascape results. However, when the enrichment analysis considered disease databases in Metascape and gProfiler, important differences were observed, as described below.

In the enrichment analysis performed using DisGeNET, the target genes of miRNAs were associated with several cancer-related terms, such as “Gallbladder Carcinoma” (log(q) = −2.6) and “Ovarian Carcinoma” (log(q) = −2.6). On the other hand, the term “Developmental delay (disorder)” (log(q) = −4.0) appeared among those with the lowest q-value in the enrichment analysis considering all target genes (Supplementary Figure S38).

Interestingly, the analysis performed by gProfiler using Human Phenotype Ontology terms returned the term “Autistic behavior” for the genes targeted by three miRNAs (hsa-miR-27a-3p: padj = 0.029; hsa-miR-221-3p: padj = 0.026; hsa-miR-19b-3p: padj = 0.047).

### 3.6. Enrichment Analysis of Target Genes Common to PBMCs, Neurons, and Sperm

As previously mentioned, PBMCs represent an important peripheral sample for studies about molecular mechanisms involved in autism, as well as for biomarker investigation. The search for possible common genetic and epigenetic signatures between PBMCs, sperm cells, and neurons can be important in its contribution to understanding the molecular characteristics of autism and its paternal inheritance. Thus, an enrichment analysis considering the miRNAs of interest that are common to these three cell types was performed.

The analysis of the target genes common to PBMCs, neurons, and sperm performed via Metascape showed that these genes are associated with mechanisms related to neurodevelopment, such as “neuron projection development” (log(q) = −1.60) and “forebrain development” (log (q) = −1.57). Terms involved in cancer also appeared as enriched, including “MicroRNAs in cancer” (log (q) = −2.05), “Signaling by ALK in cancer” (log (q) = −2.04), and “NRP1 triggered signaling pathways in pancreatic cancer” (log (q) = −1.57). Clusters defined by Metascape and the q-value are available in Table B in Table S12.

**Figure 4:**
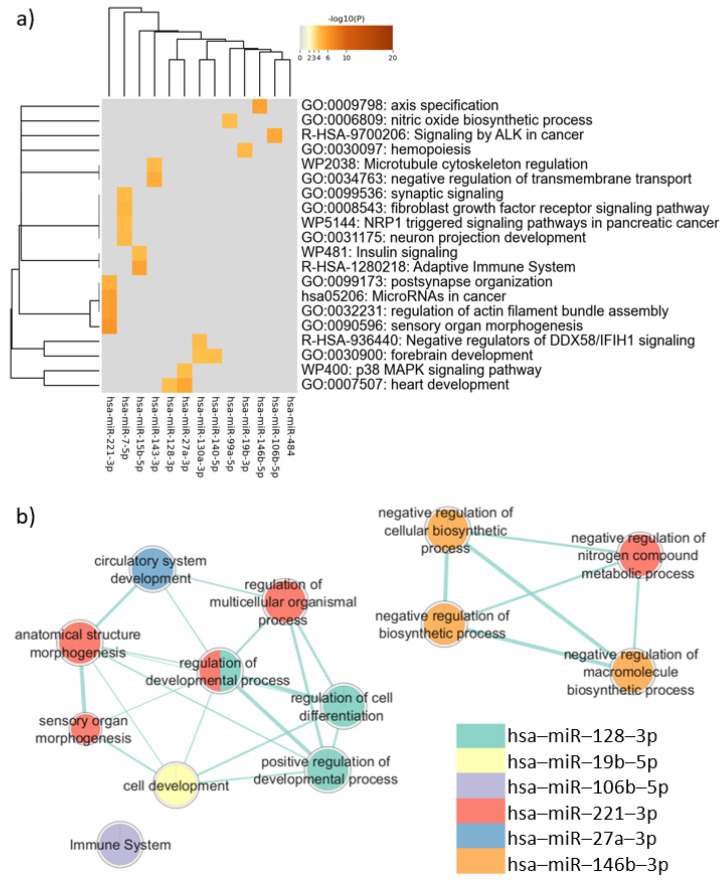
Pathway enrichment analysis for target genes expressed in common in sperm, neurons, and PBMCs. (**a**) Heatmap of enriched terms across miRNAs target genes list, produced by Metascape; (**b**) Enrichment map of enriched terms for miRNAs target genes lists created using EnrichmentMap in Cytoscape and gProfiler results.

In the enrichment analysis performed using gProfiler, only the target genes of six miRNAs were associated with biological pathways. These pathways, however, were not directly related to neurodevelopmental processes, as shown in Figure 4. The enrichment analysis considering disease databases (DisGeNET and Human Phenotype Ontology) also did not return terms related to neurodevelopmental disorders (Supplementary Figure S39).

### 3.7. Autism-Related miRNAs: Gene-to-miRNA Analysis (miRNA Enrichment)

To confirm the autism-related miRNAs that resulted from the miRNA-to-gene analysis, an inverse analysis was performed, starting from the genes reported to be involved in autism, to subsequently find the miRNAs that target them (gene-to-miRNA analysis). For this, the EnrichMir package and miRTarBase experimentally validated interactions were used.

In the analysis, using the genes from SFARI Gene or identified in GWAS studies (Table S13), 36 miRNAs were found (Table S14). None of them showed enrichment >1.5; however, hsa-miR-19a-3p (FDR = 4.96 × 10^−8^, E = 1.063), hsa-miR-6867-5p (FDR = 7.08 × 10^−6^, E = 1.044), hsa-miR-574-5p (FDR = 5.27 × 10^−5^, E = 1.024), hsa-miR-144-3p (FDR = 0.0112, E = 1.035), hsa-miR-5010- 3p (FDR = 0.0136, E = 1.136), hsa-miR-567 (FDR = 0.0157, E = 1.049), hsa-miR-18b-5p (FDR = 0.0182, E = 1.264), hsa-miR-6074 (FDR = 0.0201, E = 1.415), hsa-miR-297 (FDR = 0.0208, E = 1.029), hsa-miR-450a-2-3p (FDR = 0.0385, E = 1.262), and hsa-miR-4455 (FDR = 0.0475, E = 1.013) showed enrichment >1, which is still a good value. From the 36 miRNAs, 9 were among the 77 autism-related miRNAs expressed in the three cell types, as found in the miRNA-to-gene analysis, including hsa-miR-21-5p, hsa-miR-15b-5p, hsa-miR-181a-5p, hsa-miR-92a-3p, hsa-miR-181b-5p, hsa-miR-19b-3p, hsa-miR-744-5p, hsa-miR-221-3p, and hsa-miR-16-5p. The autism-related miRNAs that were coincident in both strategies (miRNA-to-gene and gene-to-miRNA analyses) are shown in Table 8.

In addition to the gene-to-miRNA enrichment, an analysis considering all genes involved with neurodevelopmental processes provided by the databases Gene Ontology, Reactome, and WikiPathways was also performed, since these genes are potentially involved with autism. The terms considered for this analysis were: “synapse assembly”, “synapse maturation”, “brain development”, “midbrain development”, “hindbrain development”, “forebrain development”, “generation of neurons”, and “neurogenesis” in Gene Ontology; “nervous system development” in Reactome; “ADHD and autism (ASD) linked metabolic pathways and SNP” and “Synaptic signaling pathways associated with autism spectrum disorder” in WikiPathways. In the enrichment analyses, searching for miRNAs that regulate the expression of genes involved in important pathways for synapse assembly and maturation, no miRNAs with FDR < 0.05 were identified.

In the enrichment analysis of the term “brain development” (GO:0007420), 27 miRNAs were found (Table S15). The miRNAs hsa-miR-223-3p, hsa-miR-126-3p, hsa-miR-138-5p, hsa-miR-6740-5p, hsa-miR-34a-3p, hsa-miR -6806-5p, hsa-let-7c-3p, hsa-miR-31-3p, hsa-miR-9-3p, hsa-miR-133b, hsa-miR-145-3p, hsa-miR-4260, and hsa-miR-4699-3p were the miRNAs with the highest enrichment values (E > 1.50; Table 9). Hsa-miR-21-5p (FDR = 0.0373, E = 0.754) was among the miRNAs that target genes in the “brain development” process; this miRNA is expressed in PBMCs, sperm, and neurons, and was identified as a potentially autism-related miRNA in the miRNA-to-gene analysis.

In miRNA enrichment analysis for gene ontologies, “midbrain development” (GO:0030901) and “hindbrain development” (GO:0030902), both descendant terms of “brain development” (GO:0007420), there were no miRNAs with FDR < 0.05. Nevertheless, in the enrichment analysis using all genes from the “forebrain development” pathway (GO:0030900), another “child term” of “brain development” (GO:0007420), 17 miRNAs were found (Table S16). The miRNAs hsa-miR-200c-3p, hsa-miR-34a-3p, hsa-miR-138-5p, hsa-miR-145-3p, hsa-miR-200b-3p, hsa-miR-31-3p, hsa-miR-152-3p, hsa-miR-126-3p, hsa-miR-9-3p, hsa-miR-429, hsa-miR-22-3p, hsa-miR-223-3p, and hsa-let-7c-3p were the miRNAs with the highest enrichment values (E > 1.50, Table 9). Among these miRNAs, hsa-miR-152-3p (FDR = 0.0107, E = 1.795) was also found to be expressed in PBMCs, sperm, and neurons, and it was identified as a potentially autism-related miRNA in the miRNA-to-gene analysis.

In the enrichment analysis of the biological pathway “neurogenesis” (GO: 0022008), 76 miRNAs were identified (Table S17). The miRNAs hsa-miR-223-3p, hsa-miR-1179, hsa-miR-126-3p, hsa-miR-127-3p, and hsa-miR-4632-3p were the miRNAs with the highest enrichment value (E > 1.50, Table 9). In the enrichment analysis of the biological pathway “generation of neurons” (GO: 0048699), a direct descendant of “neurogenesis” (GO: 0022008), 46 miRNAs had more target genes among the pathway genes than was expected by chance (Table S18). The miRNAs hsa-miR-223-3p, hsa-miR-1179, and hsa-miR-31-3p were the miRNAs with the highest enrichment values (E > 1.50, Table 9). Six miRNAs that regulate the expression of genes belonging to “neurogenesis” and “generation of neuron” pathways were among the 77 autism-related miRNAs that are expressed in the three cell types investigated in the current study, as shown in the miRNA-to-gene analysis: hsa-miR-140-5p, hsa-miR-21-5p, hsa-miR-148a-3p, hsa-miR-101-3p, hsa-miR-423-5p, and hsa-miR-30a-5p. Three other autism-related miRNAs were found in the gene-to-miRNA analysis (hsa-miR-127-3p, hsa-miR-378a-3p, and hsa-miR-148b-3p).

In the enrichment analysis of the Reactome biological pathway “Nervous system development” (R-HSA-9675108), 12 miRNAs were identified (Table S19). MiRNAs hsa-miR-100-5p, hsa-miR-652-3p, and hsa-miR-452-5p showed the highest enrichment values (E > 1.50, Table 9). Among the results for the “Nervous system development” biological process, 6 miRNAs were among the 77 autism-related miRNAs that are expressed in the three cell types investigated in the current study, as shown in the miRNA-to-gene analysis: hsa-miR-92a-3p, hsa-miR-744-5p, hsa-miR-16-5p, hsa-miR-100-5p, hsa-miR-186-5p, and hsa-miR-652-3p.

In the enrichment analysis of the biological pathway “Synaptic signaling pathways associated with autism spectrum disorder” (WP4539) from WikiPathways, four miRNAs were noted: hsa-miR-126-3p, hsa-miR-451a, hsa-miR-487a-3p, and hsa-miR-19a-3p (Tables S9 and S20). None of the 77 candidate miRNAs found in the miRNA-to-gene analysis were among the results of this analysis.

## 4. Discussion

Great interest in autism epigenetics has been shown since the manifestation of this condition involves the interaction between genetic and environmental factors that are biologically translated through epigenetic mechanisms. Bulk data have been generated on this subject as a consequence of the rapid evolution of next-generation sequencing technologies. However, due to the inherent complexity of autism and of genome-environment interactions, the data produced are still controversial. In view of these facts, it becomes essential to have a systematized overview of the state of the art on this topic so that subsequent studies can be conducted considering, in the best possible way, the data already available. Another complicated issue is the comprehension about if and how the mechanisms of epigenetic inheritance influence autism heredity. Thus, the goal of this study was to identify autism-related miRNAs reported by previous studies and, from this, analyze the feasibility of using these miRNAs as autism biomarkers and in the study of autism inheritance. Here, we looked at miRNAs related to autism and neurodevelopment and explored biological processes regulated by them.

By using bioinformatic tools and public genetic databases, we identified 416 miRNAs reported to be related to autism. Among these 416 autism-related miRNAs, 77 are expressed in common in PBMCs, neurons, and sperm, showing potential to be explored as biomarkers of autism and its paternal inheritance. This list was narrowed down to consider only miRNAs that have already been reported as differentially expressed in common in post-mortem brain/cerebellum and peripheral samples (blood or saliva) of autistic individuals. The 18 miRNAs obtained were considered interesting candidates as autism biomarkers. One of these miRNAs was hsa-miR-146a-5p, which showed increased expression in samples from people with autism compared to the control group in various tissues [23,26,68,77,89,90]. Indeed, the enrichment analysis of biological pathways using the genes targeted by this miRNA indicated its involvement in neurodevelopment. The 18 candidate miRNAs identified by this study target genes that have been reported as being related to autism, such as *MECP2*, *CLOCK*, *AGO1*, and *ABCA1*. According to our results, these genes are targeted by the 10 or more candidate miRNAs proposed here. Other important genes suggested as being related to autism, such as *CACNA1A* and *SLC8A1*, also appeared, although targeted by a smaller number of miRNAs. From all targeted genes, *TNRC6B* calls for special attention, since it is targeted by 16 of the 18 candidate miRNAs. Two variants of this gene showing de novo loss of function have been identified in probands according to the SFARI Gene database. However, few studies have explored the relationship of this gene with autism [91,92]. Indeed, the enrichment analysis of the biological pathways in which the genes targeted by the 18 candidate miRNAs are involved returned terms related to neurodevelopmental processes such as “brain development”, “neurogenesis”, and “synapse assembly”. Other processes that are not specifically related to autism also appeared with high frequency, such as “phosphorylation”, “cell response to stress”, and “chromatin organization”. Although not specific, these terms are undoubtedly important for neurodevelopment, since they are universal biological mechanisms.

Among the 77 autism-related miRNAs identified using the miRNA-to-genes analysis, 9 were also identified in the genes-to-miRNA analysis, and 3 of them (hsa-miR-15b-5p, hsa-miR-19b-3p, and hsa-miR-221-3p) were among the 18 candidates that are common to sperm, PBMCs, and neurons. This coincidence suggests that hsa-miR-15b-5p, hsa-miR-19b-3p, and hsa-miR-221-3p emerge as interesting targets in the search for the autism biomarkers using peripheral samples, as well as for the investigation of paternal inheritance of autism. It is relevant to observe that hsa-miR-15b-5p has the same seed sequence (AGCAGCA) present in other autism-related miRNAs (hsa-miR-15a-5p, hsa-miR-424-5p, hsa-miR-497-5p, hsa-miR-195-5p, and hsa-miR-16-5p). The seed sequence is the first two to eight nucleotides (5′ → 3′) that recognize the target mRNA and is one of the four characteristics used to predict miRNA targets [93]. This coincidence of the seed sequence suggests that miRNAs containing this seed sequence are important for autism. Furthermore, except for hsa-miR-497-5p, these miRNAs are expressed in sperm, which makes them potential targets for investigation of autism paternal inheritance. Another interesting observation is that hsa-miR-19b is the candidate miRNA with the highest number of autism-related target genes expressed in sperm and neurons, suggesting that this miRNA is an interesting marker by which to explore autism epigenetic inheritance. In addition, this miRNA is the second one with the highest number of target genes that are common to the three cell types considered for this study. A study on paternal epigenetic inheritance of miRNA related to autism traits in mice showed differential expression of miR-19b in blood and sperm [12,94]. Although these studies with animal models indicate a role of miRNA in epigenetic inheritance, it is a very complicated issue to be explored in humans. Still, the investigation of miRNA-mediated epigenetic inheritance through sperm is promising due to the practicality of sample collection and high cell number. Our analyses reinforce that the study of miRNA participation in the paternal inheritance of autism is worthwhile.

The available genetic databases contain a great and valuable amount of data on miRNAs and genes linked to the most diverse biological processes and diseases. However, the diversity of techniques and methods adopted in the studies used to build these databases inevitably leads to differences in the results obtained by these studies, which can be considered a limitation for downstream studies that use these databases. The variety of samples, the different statistical tests, the age of participants, the tools applied to diagnose autism, and the different methodologies to measure miRNA expression can produce discrepant results that can make it difficult to interpret and compare the data obtained by these studies. Another issue to be considered is the phenotypic variability observed among people with autism, who can manifest symptoms in different ways and require different degrees of support. There are also limitations concerning target gene prediction and pathway enrichment analysis, such as concerns about the presence of too many false targets in miRNA target prediction tools [95], as well as biases in results toward more studied biological pathways, especially those implicated in cancer [96]. In the current study, enrichment analyses results, especially the disease enrichment analysis performed by Metascape, identified different types of cancer. Previous studies have suggested that autism and cancer may share similar biological pathways [97,98,99,100], which suggests that the same mechanisms may contribute to the development of both conditions. Nevertheless, it is necessary to highlight that there are biases in the enrichment analysis for pathways involved in cancer. Therefore, care must be taken when interpreting enrichment analysis results. Another important issue was the fact that the quality of the studies from which all data were extracted was not technically assessed. This criterion was adopted due to the high variability of the studies and also because subsequent restriction tools were applied, aiming to exclude weak data. It must also be considered that most of the genes included were obtained from the SFARI gene, a database that contains genes suggested as candidates for autism identified through genetic association studies, genes related to syndromic autism, and genes in which rare mutations have been found. In this context, genes related to the term “syndromic autism” refer to genes with mutations that are associated with an increased risk and related to additional characteristics not necessary for an ASD diagnosis. Recent discussions about the use of the term “syndromic” call attention to the different interpretations of this term [101], which has implications for the way by which it is included in databases. Furthermore, possible influences of the deregulation of miRNAs on these genes would affect their expression and would not, therefore, have the same effect as the variants described.

Despite these issues, the large amount of data and the evidence on the relationship between miRNA expression and autism are encouraging, so bioinformatic tools are useful to explore the bulk data already available. Because different genetic databases and multiple algorithms for bioinformatic analyses are available, the use of different strategies and tools to investigate miRNAs related to autism seems fundamental. Indeed, our data indicate that the use of different tools is important to provide more complete data, as indicated by Metascape versus gProfiler/clusterMaker2 analyses, that showed similar but not identical results. Although most of the data of Metascape differ from those of clusterMaker2, some results coincided, showing some convergence of biological pathways generated by both platforms. Similarly, the gene-to-miRNA analysis produced different results from miRNA-to-gene analysis, although some coincidence was observed between the two strategies.

In general, this study brought together the available information about miRNAs and genes potentially related to autism. More importantly, it was possible to pinpoint miRNAs that emerge as candidate biomarkers for autism and its paternal inheritance. Experimental studies are in progress, aiming to validate these data. Finally, although it is an inherent factor in any study, it is important to mention that the parameters adopted for searching and analyzing miRNAs and genes restrict the results generated according to the capabilities of the software used in the analysis. Experimental studies are necessary to validate these data.

## 5. Conclusions

The data obtained in the current study revealed that there is no consensus concerning miRNAs related to autism. However, the identification of coincident miRNAs with altered expression in different studies and databases indicates that the use of miRNAs as autism biomarkers is promising. Furthermore, the coincidence in the expression of miRNAs in neurons, PBMCs, and sperm pointed to a group of miRNAs potentially related to autism, suggesting that they could be used as candidate biomarkers for autism and its paternal inheritance.

## Figures and Tables

**Table 1:** Parameters adopted in the EnrichmentMap app using Cytoscape.

Enrichment Analysis	Biological Pathways Enrichment Map	Human Phenotypes Ontology Enrichment Map
(a) all identified target genes of each miRNA	OC = 0.5	JC = 0.3
(b) target genes expressed in common in sperm and neurons	OC = 0.5	OC = 0.5
(c) target genes expressed in common in PBMCs, sperm, and neurons	OC = 0.5	OC = 0.5

OC: overlap coefficient; JC: Jaccard coefficient.

**Table 2:** Autism-related miRNAs only expressed in one cell type (PBMCs, neurons, or sperm).

Sperm	Neurons	PBMCs
hsa-miR-424-5p	hsa-miR-432-5p	hsa-miR-451a
hsa-miR-619-5p	hsa-miR-196a-5p	hsa-miR-320a-3p
hsa-miR-193b-3p	hsa-miR-494-3p	hsa-miR-144-3p
		hsa-miR-664a-3p

**Table 3:** Autism-related miRNAs expressed in common in two cell types.

Sperm and Neurons	Sperm and PBMCs	Neurons and PBMCs
hsa-miR-106a-5p	hsa-miR-223-3p	hsa-miR-874-3p
hsa-miR-379-5p	hsa-miR-142-3p	hsa-miR-199a-5p
hsa-miR-148a-5p	hsa-miR-142-5p	
	hsa-miR-146a-5p	
	hsa-miR-15a-5p	
	hsa-miR-29a-3p	
	hsa-miR-29b-3p	
	hsa- miR-29c-3p,	
	hsa-miR-19a-3p	
	hsa-miR-155-5p	

**Table 4:** Autism-related miRNAs expressed in sperm according to SpermBase and miRmine.

miRNA	
hsa-miR-106a-5p	hsa-miR-7-5p
hsa-miR-223-3p	hsa-let-7a-5p
hsa-miR-142-3p	hsa-miR-128-3p
hsa-miR-146a-5p	hsa-miR-99a-5p
hsa-miR-155-5p	hsa-miR-15b-5p
hsa-miR-15a-5p	hsa-miR-484
hsa-miR-29b-3p	hsa-miR-19b-3p
hsa-miR-29c-3p	hsa-miR-106b-5p
hsa-miR-19a-3p	hsa-miR-221-3p
hsa-miR-29a-3p	hsa-miR-574-3p
hsa-miR-424-5p	hsa-miR-93-5p
hsa-miR-619-5p	hsa-miR-27a-3p
hsa-miR-143-3p	hsa-miR-335-3p
hsa-miR-140-5p	hsa-miR-130a-3p
hsa-miR-23a-3p	hsa-miR-146b-5p

**Table 5:** Autism-related miRNAs included in the pathway enrichment analysis.

miRNA	References
hsa-let-7a-5p	[25] (blood); [68] (brain); [69] (saliva)
hsa-miR-93-5p	[70] (cerebellar cortex); [27] (monocytes); [71] (serum)
hsa-miR-27a-3p	[70] (cerebellar cortex); [72] (serum); [73] (saliva); [74] (blood)
hsa-miR-146b-5p	[70] (cerebellar cortex); [75] (saliva); [76] (brain)
hsa-miR-140-5p	[70] (cerebellar cortex); [76] (brain); [77] (saliva)
hsa-miR-23a-3p	[70] (cerebellar cortex); [73] (saliva); [78] (brain); [79] (saliva); [80] (brain); [81] (serum)
hsa-miR-7-5p	[70] (cerebellar cortex); [68] (brain); [73] (saliva); [71] (serum); [79] (saliva); [82] (plasma)
hsa-miR-15b-5p	[70] (cerebellar cortex); [25] (blood); [83] (blood)
hsa-miR-484	[70] (cerebellar cortex); [78] (brain); [84] (plasma)
hsa-miR-19b-3p	[72] (serum); [25] (blood); [68] (brain); [85] (serum)
hsa-miR-106b-5p	[70] (cerebellar cortex); [72] (serum); [86] (serum); [87] (serum); [82] (plasma)
hsa-miR-221-3p	[78] (brain); [80] (brain); [82] (plasma)
hsa-miR-574-3p	[25] (blood); [71] (serum); [76] (brain)
hsa-miR-130a-3p	[72] (serum); [76] (brain)
hsa-miR-335-3p	[73] (saliva); [78] (brain)
hsa-miR-143-3p	[80] (brain); [77] (saliva)
hsa-miR-128-3p	[70] (cerebellar cortex); [88] (serum)
hsa-miR-99a-5p	[80] (brain); [82] (plasma)

**Table 6:** Number of genes targeted by autism-related candidate miRNAs.

miRNA	Total	Sperm and Neurons	Sperm, Neurons, and PBMCs	Target Genes Related to Autism		
				Total	Sperm and Neurons	Sperm, Neurons, and PBMCs
hsa-miR-335-3p	2549	822	488	292	120	54
hsa-miR-93-5p	2017	754	476	227	110	57
hsa-miR-106b-5p	2002	720	456	211	97	49
hsa-miR-15b-5p	1898	715	451	219	100	59
hsa-miR-484	1861	749	534	196	102	72
hsa-miR-27a-3p	1827	655	390	210	102	52
hsa-miR-128-3p	1807	636	379	201	104	52
hsa-miR-23a-3p	1726	574	359	191	92	48
hsa-miR-7-5p	1694	623	393	186	96	54
hsa-miR-19b-3p	1675	630	392	243	125	61
hsa-let-7a-5p	1585	539	338	152	75	37
hsa-miR-143-3p	1478	557	334	185	97	49
hsa-miR-221-3p	1443	559	373	186	92	53
hsa-miR-130a-3p	1351	501	316	169	73	39
hsa-miR-146b-5p	1331	489	299	161	80	39
hsa-miR-140-5p	1229	487	308	181	99	50
hsa-miR-99a-5p	1037	405	263	103	60	30
hsa-miR-574-3p	954	345	213	100	49	23

**Table 7:** Number of biological pathways related to the nervous system or neurodevelopment associated with the genes targeted by the miRNAs of interest.

miRNA	Metascape	gProfiler and EnrichmentMap
hsa-miR-335-3p	2	5
hsa-miR-93-5p	1	3
hsa-miR-106b-5p	3	3
hsa-miR-15b-5p	3	4
hsa-miR-484	1	1
hsa-miR-27a-3p	2	3
hsa-miR-128-3p	3	3
hsa-miR-23a-3p	0	3
hsa-miR-7-5p	2	4
hsa-miR-19b-3p	1	2
hsa-let-7a-5p	1	1
hsa-miR-143-3p	2	2
hsa-miR-221-3p	3	1
hsa-miR-130a-3p	2	2
hsa-miR-146b-5p	5	4
hsa-miR-140-5p	3	4
hsa-miR-99a-5p	0	0
hsa-miR-574-3p	0	1

**Table 8:** Autism-related miRNAs found via miRNA-to-gene and via gene-to miRNA strategies.

miRNA	FDR	Enrichment
has-miR-19b-3p	4.96 × 10^−8^	0.97
has-miR-15b-5p	0.00035	0.727
hsa-miR-21-5p	0.000353	0.787
hsa-miR-16-5p	0.00724	0.447
hsa-miR-744-5p	0.0126	0.77
hsa-miR-181a-5p	0.0149	0.678
hsa-miR-92a-3p	0.0165	0.432
has-miR-221-3p	0.0337	0.749
hsa-miR-181b-5p	0.0385	0.741

**Table 9:** miRNA enrichment analysis of genes involved with neurodevelopment or autism according to Gene Ontology, WikiPathways, or Reactome.

miRNA	Overlap	Enrichment	FDR
Brain development (GO:0007420)			
hsa-miR-223-3p	19	1.839	0.001298270972
hsa-miR-126-3p	13	2.075	0.003057508899
hsa-miR-138-5p	18	1.617	0.007269372947
hsa-miR-6740-5p	11	2.103	0.007269372947
hsa-miR-34a-3p	14	1.734	0.0116666655
hsa-miR-6806-5p	10	2.018	0.01729235795
hsa-let-7c-3p	14	1.634	0.02040906023
hsa-miR-31-3p	12	1.756	0.02282151648
hsa-miR-9-3p	14	1.593	0.0245245798
hsa-miR-133b	12	1.639	0.03731966297
hsa-miR-145-3p	11	1.668	0.04186509355
hsa-miR-4260	11	1.686	0.04186509355
hsa-miR-4699-3p	12	1.591	0.04186509355
Forebrain development (GO:0030900)			
hsa-miR-200c-3p	22	1.96	7.98 × 10^−5^
hsa-miR-34a-3p	12	2.253	0.004230976391
hsa-miR-138-5p	13	1.909	0.009118847038
hsa-miR-145-3p	10	2.259	0.009118847038
hsa-miR-200b-3p	16	1.696	0.009118847038
hsa-miR-31-3p	10	2.226	0.009118847038
hsa-miR-152-3p	14	1.795	0.01067551065
hsa-miR-126-3p	9	2.273	0.01121765579
hsa-miR-9-3p	11	1.995	0.01339422516
hsa-miR-429	13	1.69	0.02892920941
hsa-miR-22-3p	14	1.604	0.03007451519
hsa-miR-223-3p	11	1.814	0.0332969535
hsa-let-7c-3p	10	1.9	0.03376666551
Neurogenesis (GO: 0022008)			
hsa-miR-223-3p	30	1.641	8.01 × 10^−6^
hsa-miR-1179	17	1.737	0.001535995179
hsa-miR-126-3p	16	1.542	0.007377225259
hsa-miR-127-3p	9	1.791	0.02587966401
hsa-miR-4632-3p	9	1.627	0.04208567062
Generation of neurons (GO: 0048699)			
hsa-miR-223-3p	28	1.756	1.27 × 10^−5^
hsa-miR-1179	15	1.767	0.003310869485
hsa-miR-31-3p	16	1.54	0.01157149572
Nervous system development (R-HSA-9675108)			
hsa-miR-100-5p	28	1.599	0.0001058059054
hsa-miR-652-3p	17	1.569	0.01202419931
hsa-miR-452-5p	11	1.945	0.01686604689
Synaptic signaling pathways associated with autism spectrum disorder (WP4539)			
hsa-miR-126-3p	6	3.78	0.0001311122431
hsa-miR-451a	4	3.458	0.01312953894
hsa-miR-487a-3p	4	3.325	0.02304742794
hsa-miR-19a-3p	9	2.228	0.02807879676

FDR < 0.05, E > 1.5.

## Data Availability

The datasets generated during the current study are available as Supplementary Materials, as well the script used for miRNA enrichment analysis.

## Supplementary Materials

The following supporting information can be downloaded at: https://www.mdpi.com/article/10.3390/genes16040418/s1, Table S1. Search strategies used to find autism-related miRNAs; Table S2: Biological pathways selected for miRNA enrichment analysis; Table S3: Genes from biological pathways selected for miRNA enrichment analysis; Table S4: miRNAs found in the literature search; Table S5: miRNAs found in the literature search and that are expressed in PBMCs, neurons and sperm; Table S6: Genes targeted by the 18 candidate miRNA; Table S7: Complete Metascape results for all genes targeted by the 18 candidate miRNA; Table S8: Clusters defined by clusterMaker2 using gProfiler results for all genes targeted by the 18 candidate miRNA; Table S9: Clusters created by clusterMaker2 for target genes of each miRNA individually; Table S10: Clusters defined by clusterMaker2 using gProfiler results for Human Phenotype Ontology for all genes targeted by the 18 candidate miRNA; Table S11: Enrichment analysis of target genes common to neurons and sperm performed using Metascape; Table S12: Enrichment analysis of target genes common to PBMCs, neurons and sperm performed using Metascape; Table S13: Autism-related genes from SFARI Gene or identified in GWAS studies; Table S14: Results of miRNA enrichment analysis using autism-related genes; Table S15: Results of miRNA enrichment analusis using genes from “brain development” (GO:0007420); Table S16: Results of miRNA enrichment analusis using genes from “forebrain development” pathway (GO:0030900); Table S17: Results of miRNA enrichment analusis using genes from “neurogenesis” (GO: 0022008); Table S18: Results of miRNA enrichment analusis using genes from “generation of neurons” (GO: 0048699); Table S19: Results of miRNA enrichment analusis using genes from “Nervous system development” (R-HSA-9675108); Table S20: Results of miRNA enrichment analusis using genes from “Synaptic signaling pathways associated with autism spectrum disorder” (WP4539); Figures S1–S39: Enrichment analysis results; File S1: R script used for miRNA enrichment analysis.

## Abbreviations

The following abbreviations are used in this manuscript:

ADHD	Attention deficit hyperactivity disorder
ASD	Autism spectrum disorder
E	Enrichment value
eQTL	Expression quantitative trait loci
FDR	False discovery rate
GO	Gene Ontology
GWAS	Genome wide association studies
miRNA	microRNA
Padj	Ajusted *p*-value
PBMC	Peripheral blood mononuclear cells
PCR	Polymerase chain reaction
RPM	Reads per million
SFARI	Simons Foundation Autism Research Initiative
sncRNA	small non-coding RNA
SNP	Single nucleotide polymorphisms
