# Supplementary material for: Bioinformatic Analysis of Autism-Related miRNAs and Their PoTential as Biomarkers for Autism Epigenetic Inheritance
Source: Genes (Basel). 2025 Mar 31;16(4):418. doi: 10.3390/genes16040418 (PMC12026732; doi:10.3390/genes16040418)
Supplement: Supplementary file 1 [file genes-16-00418-s001.zip › genes-3497345-supplementary/Figure S1.pdf]

Supplementary figures – Figure S1

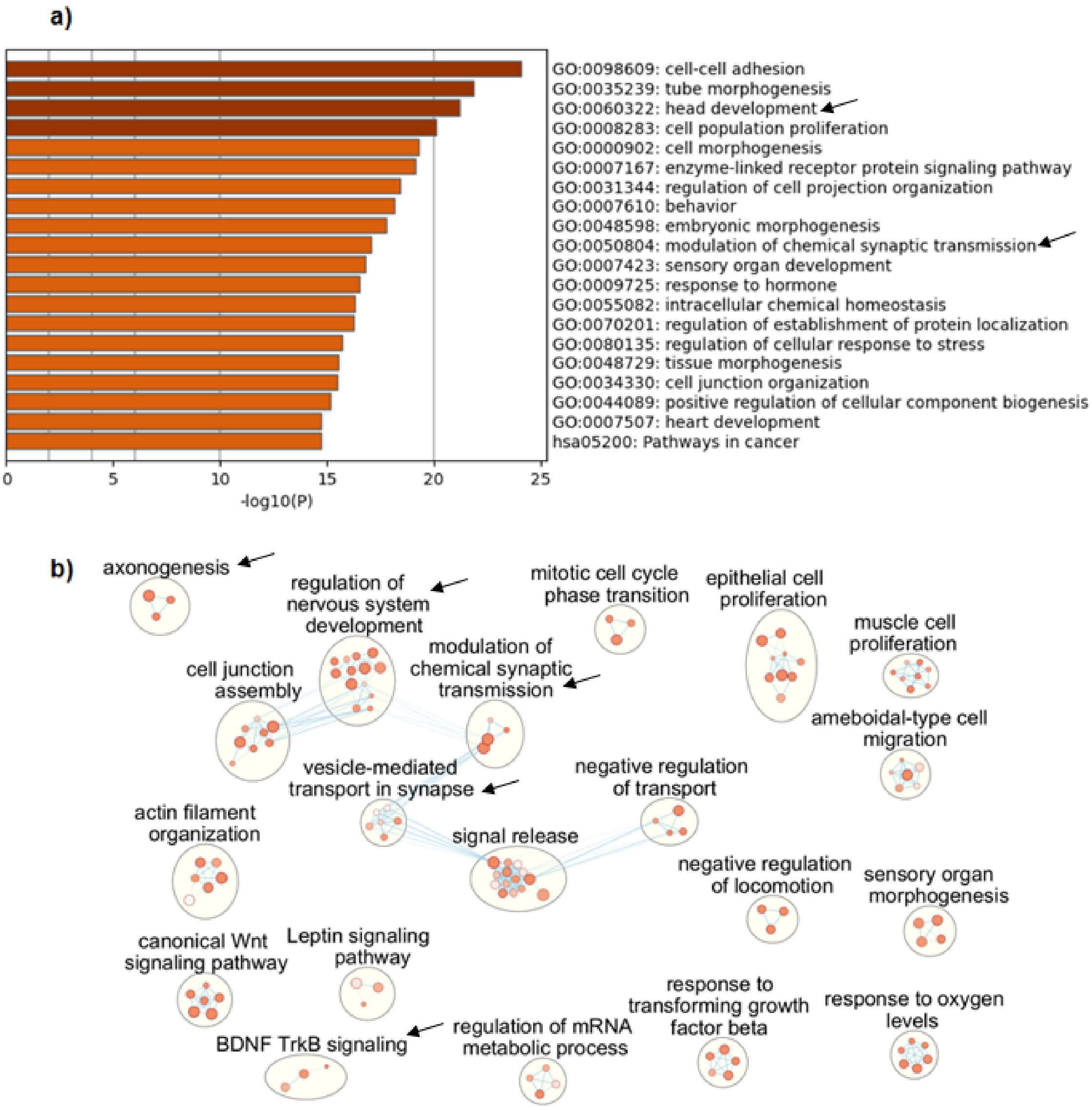

Supplementary figure 1. Pathway enrichment analysis results for genes targeted by hsa-miR-335-3p. a) Bar graph of enriched terms found in Metascape analysis, colored by p-values; b) Clusters defined by clusterMaker2 based on gProfiler results. Arrows indicate clusters related to nervous system or neurodevelopment.

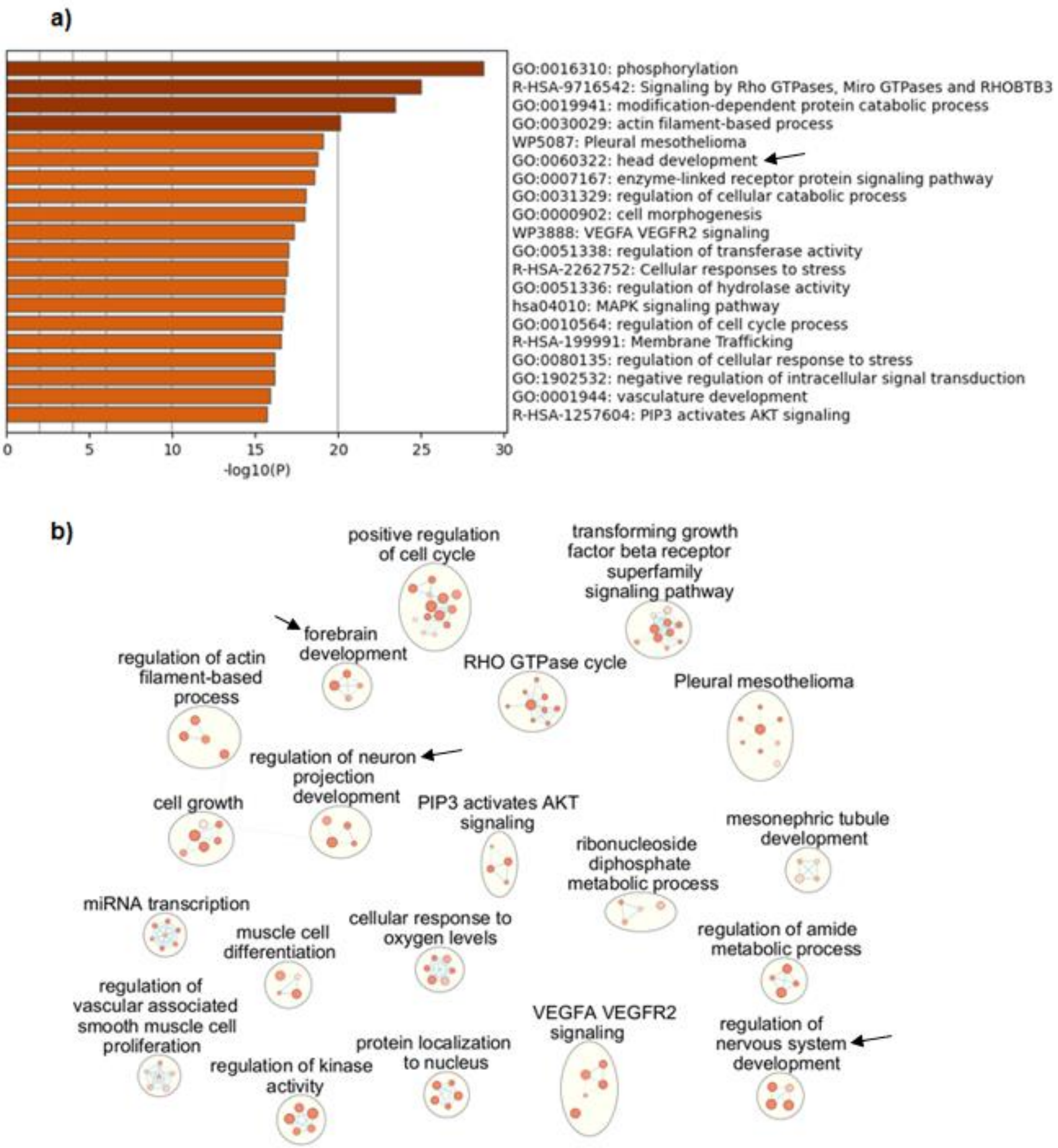

Supplementary figure 2. Pathway enrichment analysis results for genes targeted by hsa-miR-93-5p. a) Bar graph of enriched terms found in Metascape analysis, colored by p-values; b) Clusters defined by clusterMaker2 based on gProfiler results. Arrows indicate clusters related to nervous system or neurodevelopment.

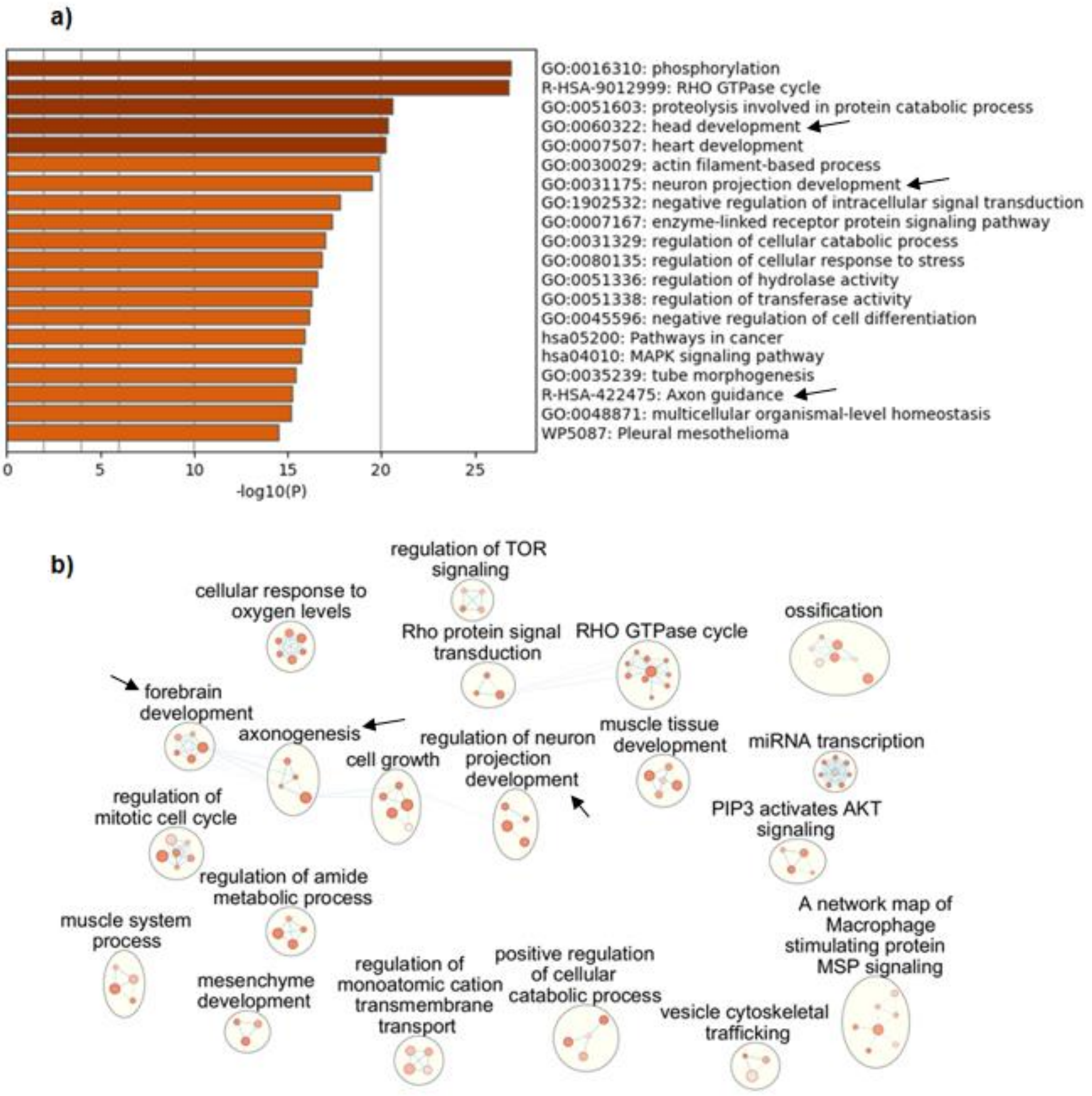

Supplementary figure 3. Pathway enrichment analysis results for genes targeted by hsa-miR-106b-5p. a) Bar graph of enriched terms found in Metascape analysis, colored by p-values; b) Clusters defined by clusterMaker2 based on gProfiler results. Arrows indicate clusters related to nervous system or neurodevelopment.

a)

b)

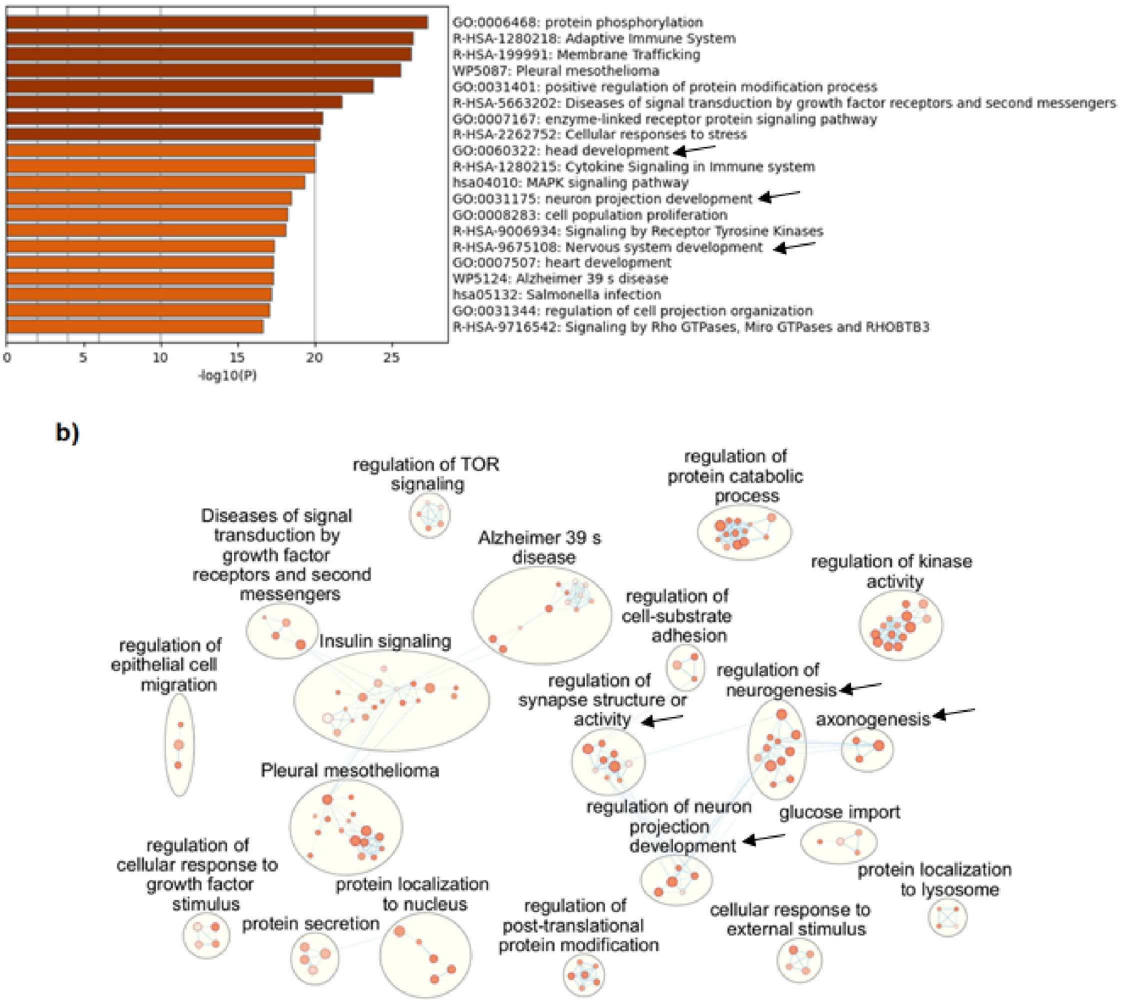

Supplementary figure 4. Pathway enrichment analysis results for genes targeted by hsa-miR-15b-5p. a) Bar graph of enriched terms found in Metascape analysis, colored by p-values; b) Clusters defined by clusterMaker2 based on gProfiler results. Arrows indicate clusters related to nervous system or neurodevelopment.

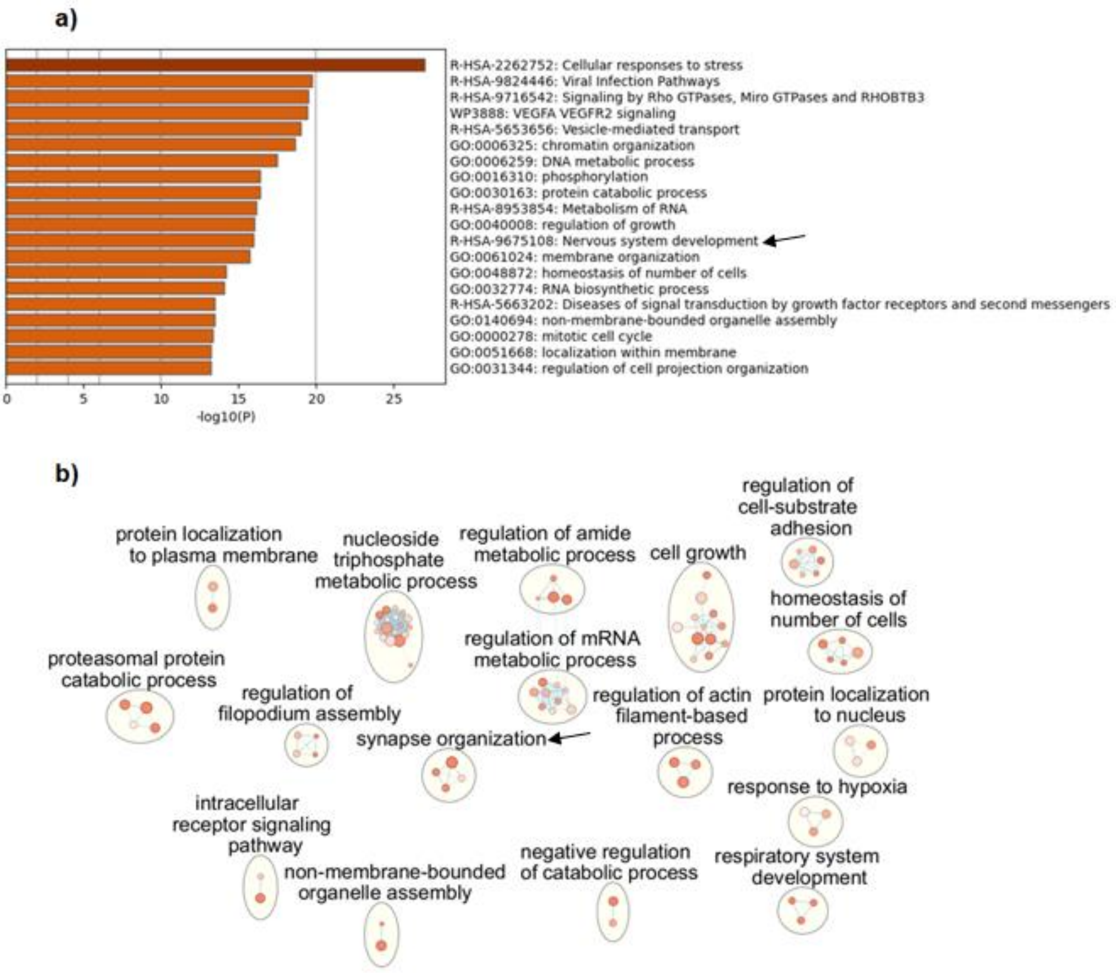

Supplementary figure 5. Pathway enrichment analysis results for genes targeted by hsa-miR-484. a) Bar graph of enriched terms found in Metascape analysis, colored by p-values; b) Clusters defined by clusterMaker2 based on gProfiler results. Arrows indicate clusters related to nervous system or neurodevelopment.

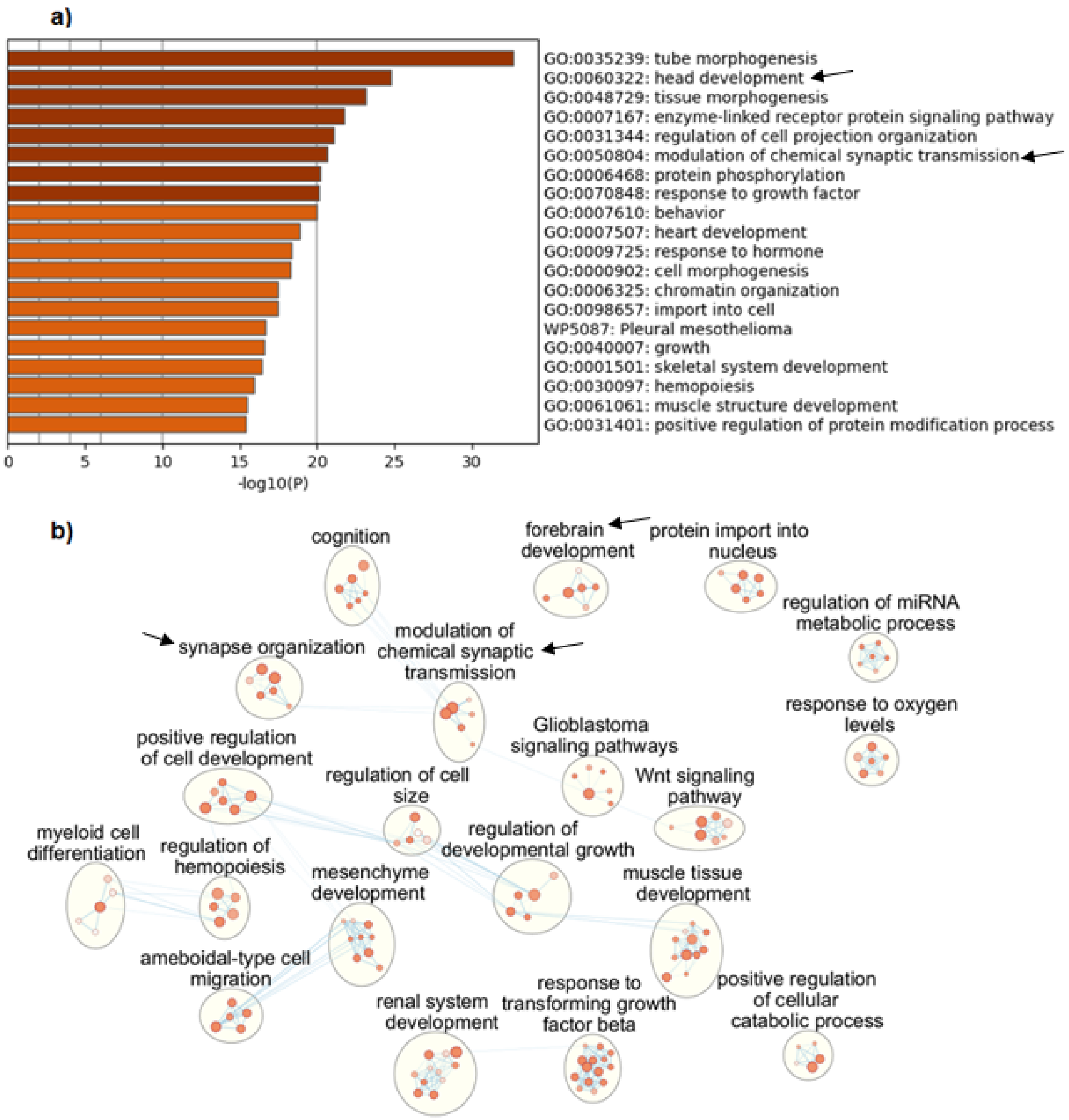

Supplementary figure 6. Pathway enrichment analysis results for genes targeted by hsa-miR-27a-3p. a) Bar graph of enriched terms found in Metascape analysis, colored by p-values; b) Clusters defined by clusterMaker2 based on gProfiler results. Arrows indicate clusters related to nervous system or neurodevelopment.

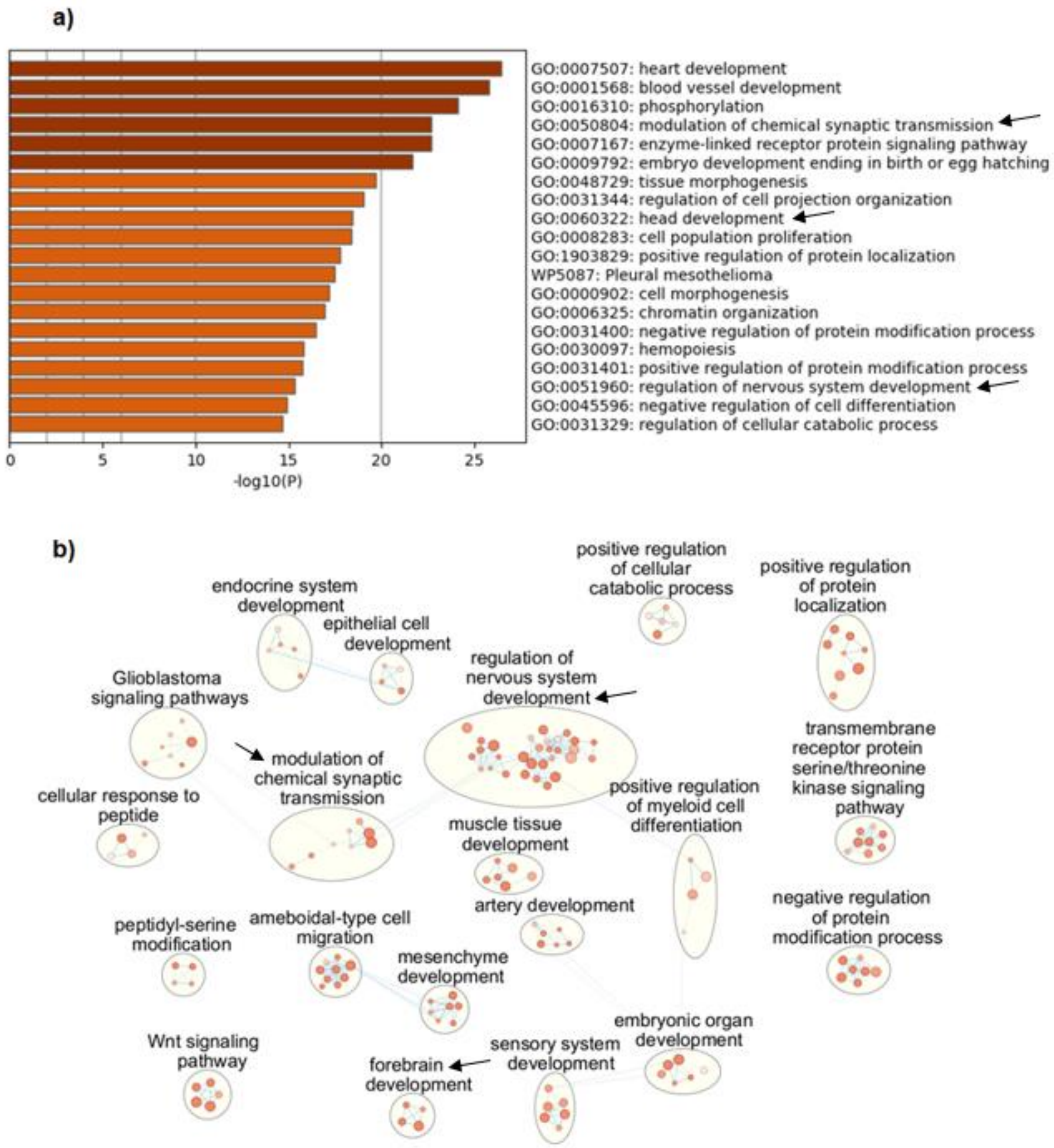

Supplementary figure 7. Pathway enrichment analysis results for genes targeted by hsa-miR-128-3p. a) Bar graph of enriched terms found in Metascape analysis, colored by p-values; b) Clusters defined by clusterMaker2 based on gProfiler results. Arrows indicate clusters related to nervous system or neurodevelopment.

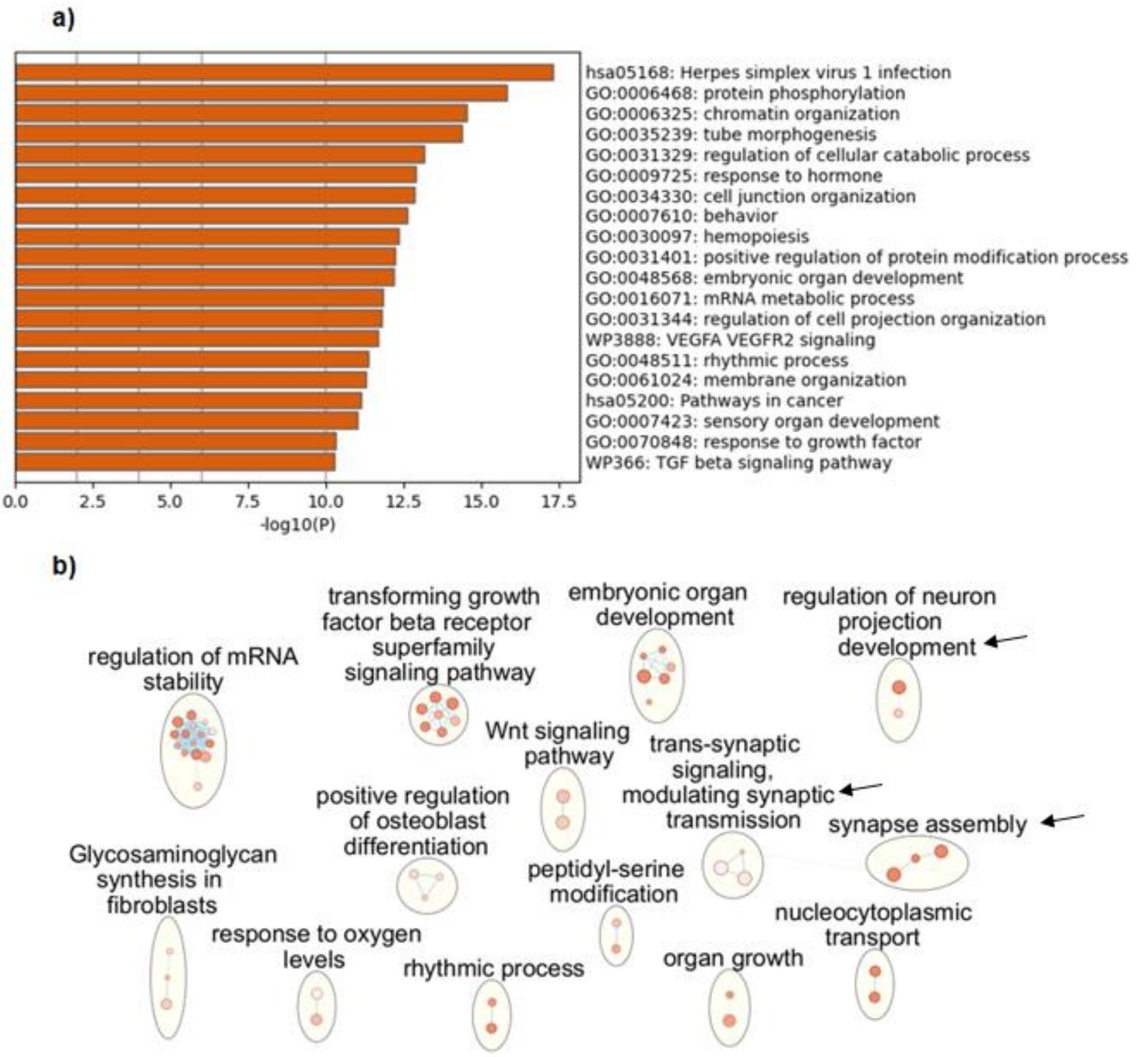

Supplementary figure 8. Pathway enrichment analysis results for genes targeted by hsa-miR-23a-3p. a) Bar graph of enriched terms found in Metascape analysis, colored by p-values; b) Clusters defined by clusterMaker2 based on gProfiler results. Arrows indicate clusters related to nervous system or neurodevelopment.

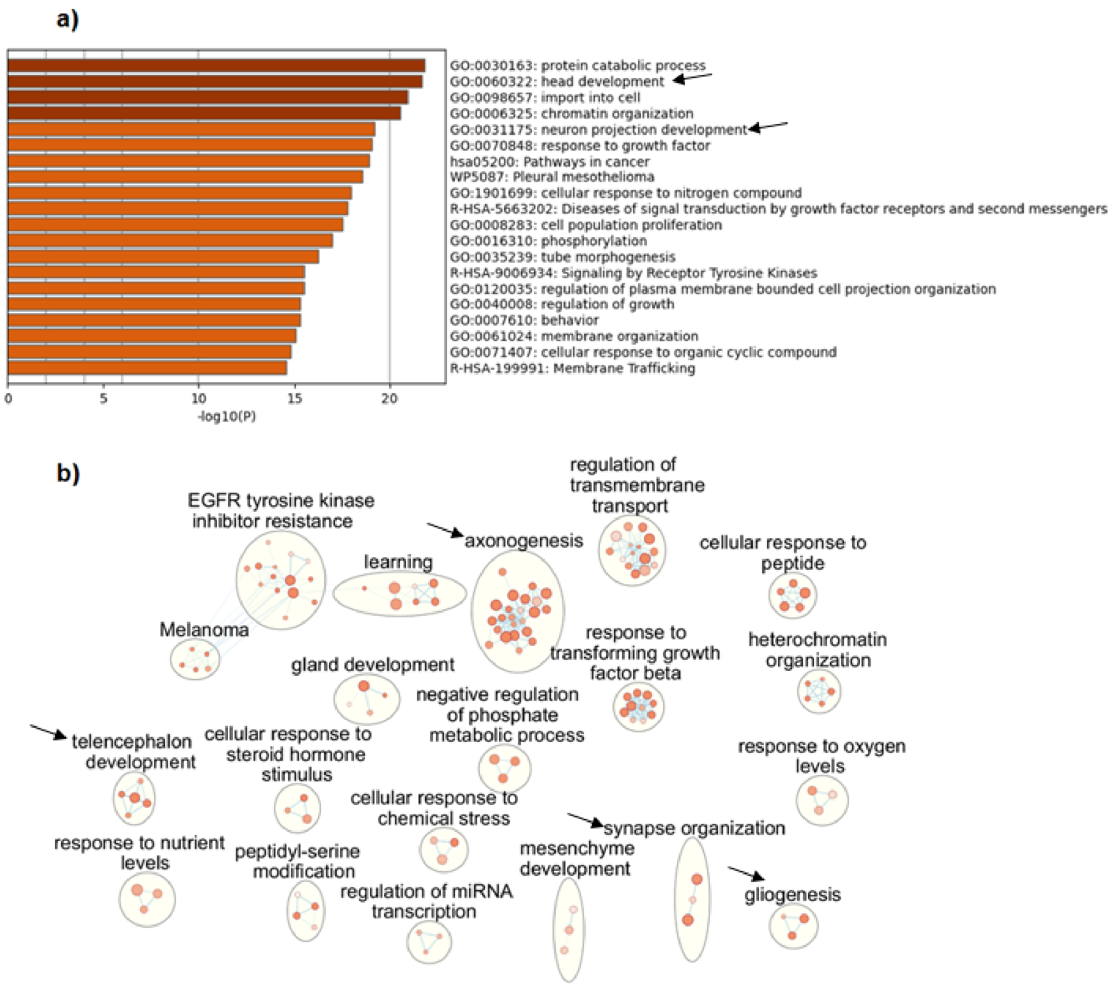

Supplementary figure 9. Pathway enrichment analysis results for genes targeted by hsa-miR-7-5p. a) Bar graph of enriched terms found in Metascape analysis, colored by p-values; b) Clusters defined by clusterMaker2 based on gProfiler results. Arrows indicate clusters related to nervous system or neurodevelopment.

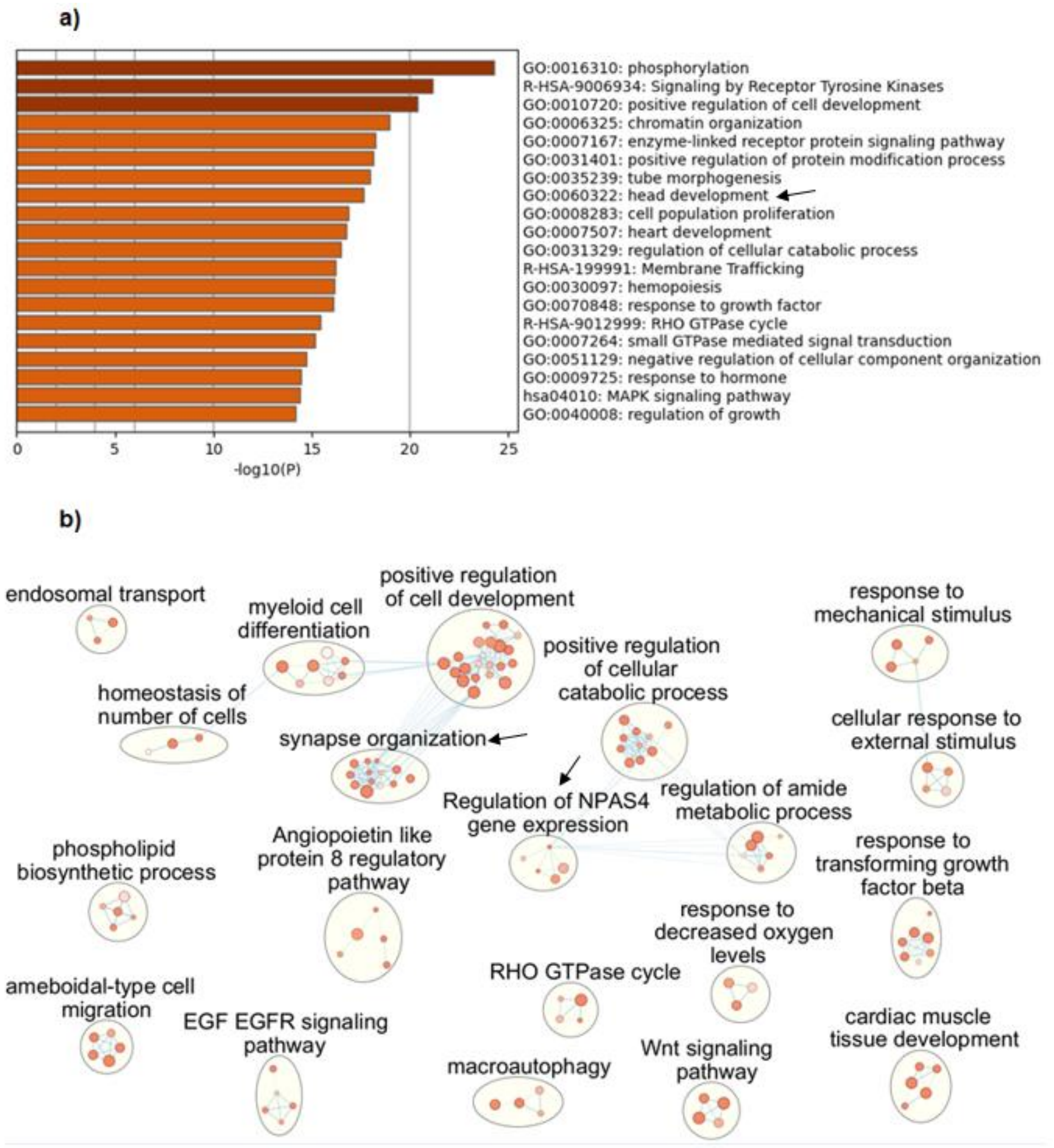

Supplementary figure 10. Pathway enrichment analysis results for genes targeted by hsa-miR-19b-3p. a) Bar graph of enriched terms found in Metascape analysis, colored by p-values; b) Clusters defined by clusterMaker2 based on gProfiler results. Arrows indicate clusters related to nervous system or neurodevelopment.

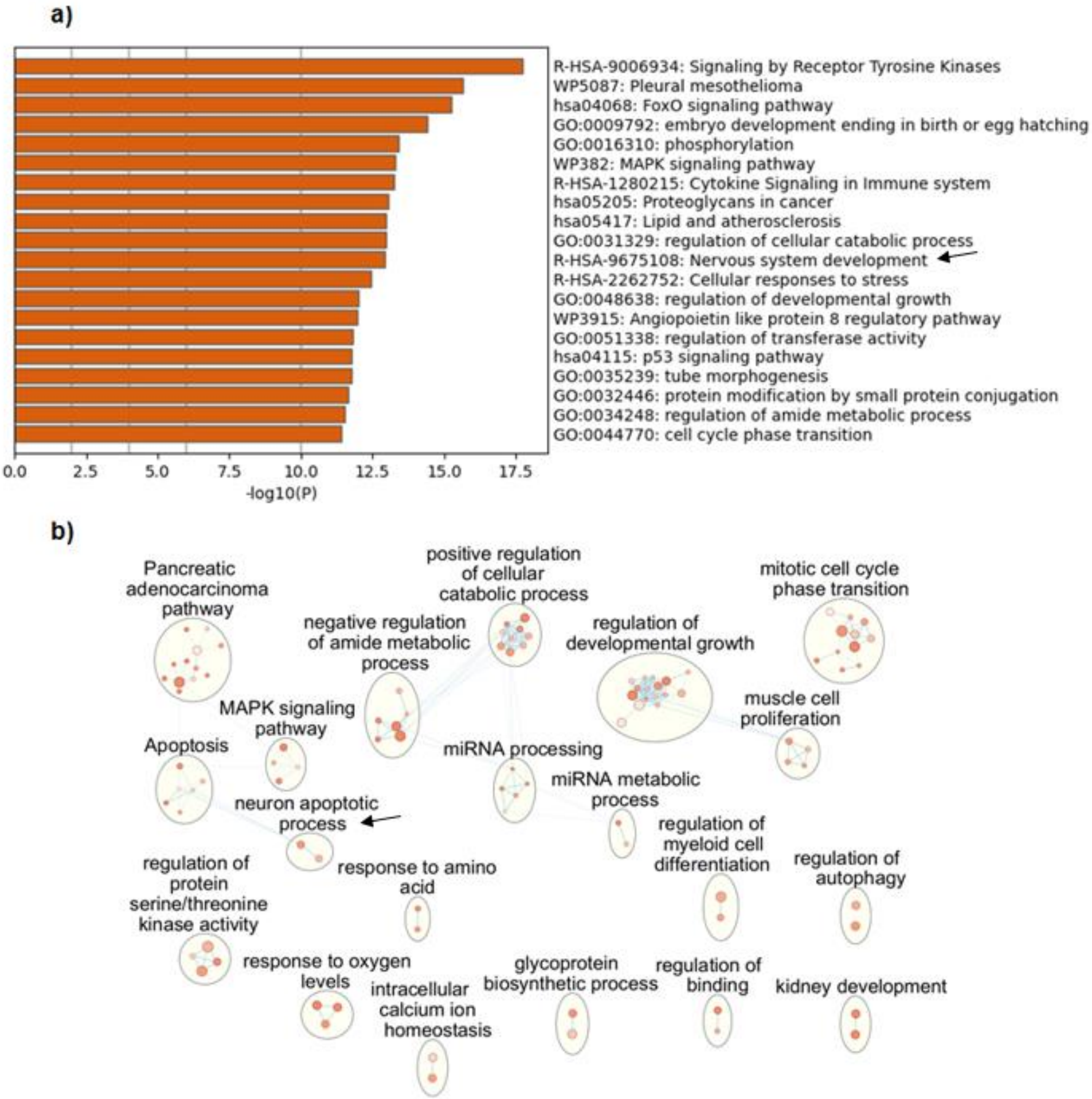

Supplementary figure 11. Pathway enrichment analysis results for genes targeted by hsa-let-7a-5p. a) Bar graph of enriched terms found in Metascape analysis, colored by p-values; b) Clusters defined by clusterMaker2 based on gProfiler results. Arrows indicate clusters related to nervous system or neurodevelopment.

a)

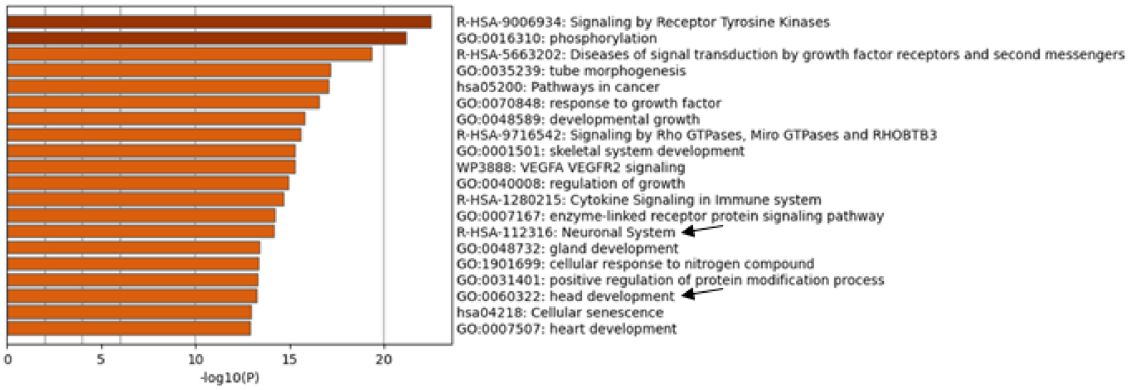

b)

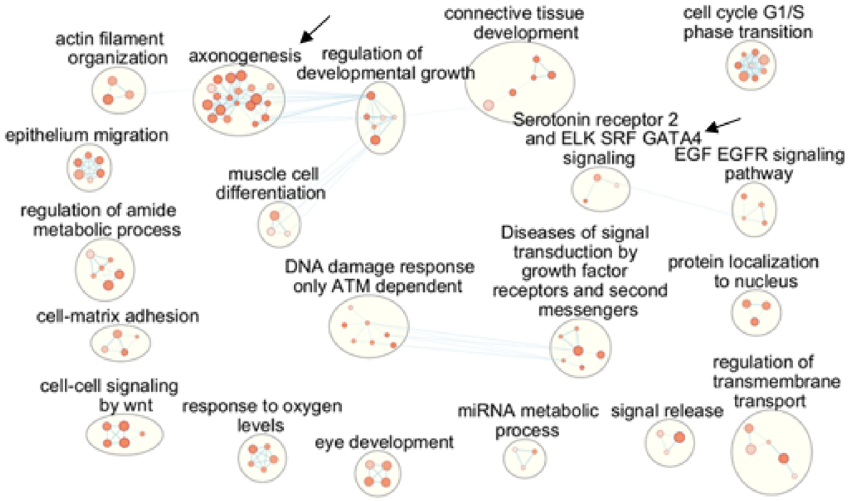

Supplementary figure 12. Pathway enrichment analysis results for genes targeted by hsa-miR-143-3p. a) Bar graph of enriched terms found in Metascape analysis, colored by p-values; b) Clusters defined by clusterMaker2 based on gProfiler results. Arrows indicate clusters related to nervous system or neurodevelopment.

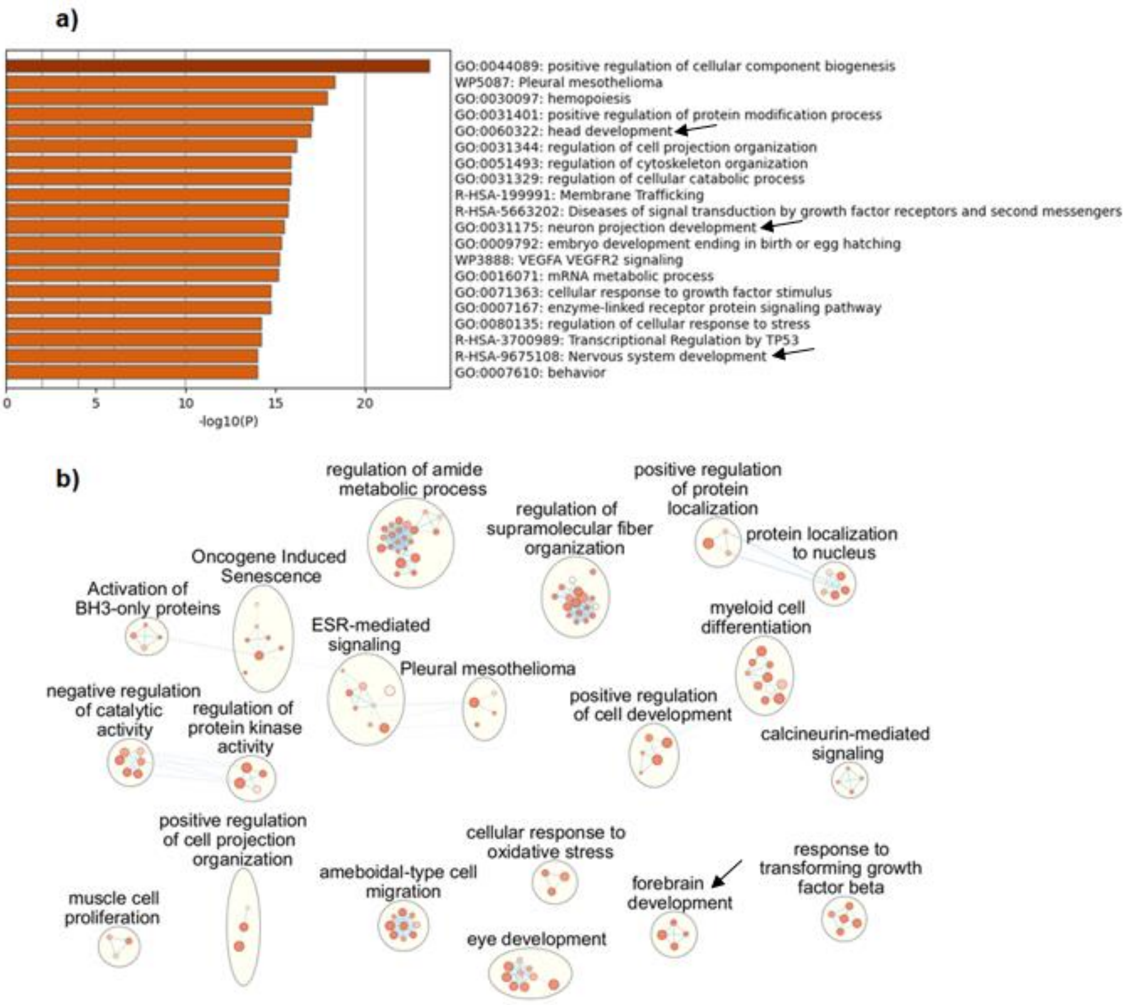

Supplementary figure 13. Pathway enrichment analysis results for genes targeted by hsa-miR-221-3p. a) Bar graph of enriched terms found in Metascape analysis, colored by p-values; b) Clusters defined by clusterMaker2 based on gProfiler results. Arrows indicate clusters related to nervous system or neurodevelopment.

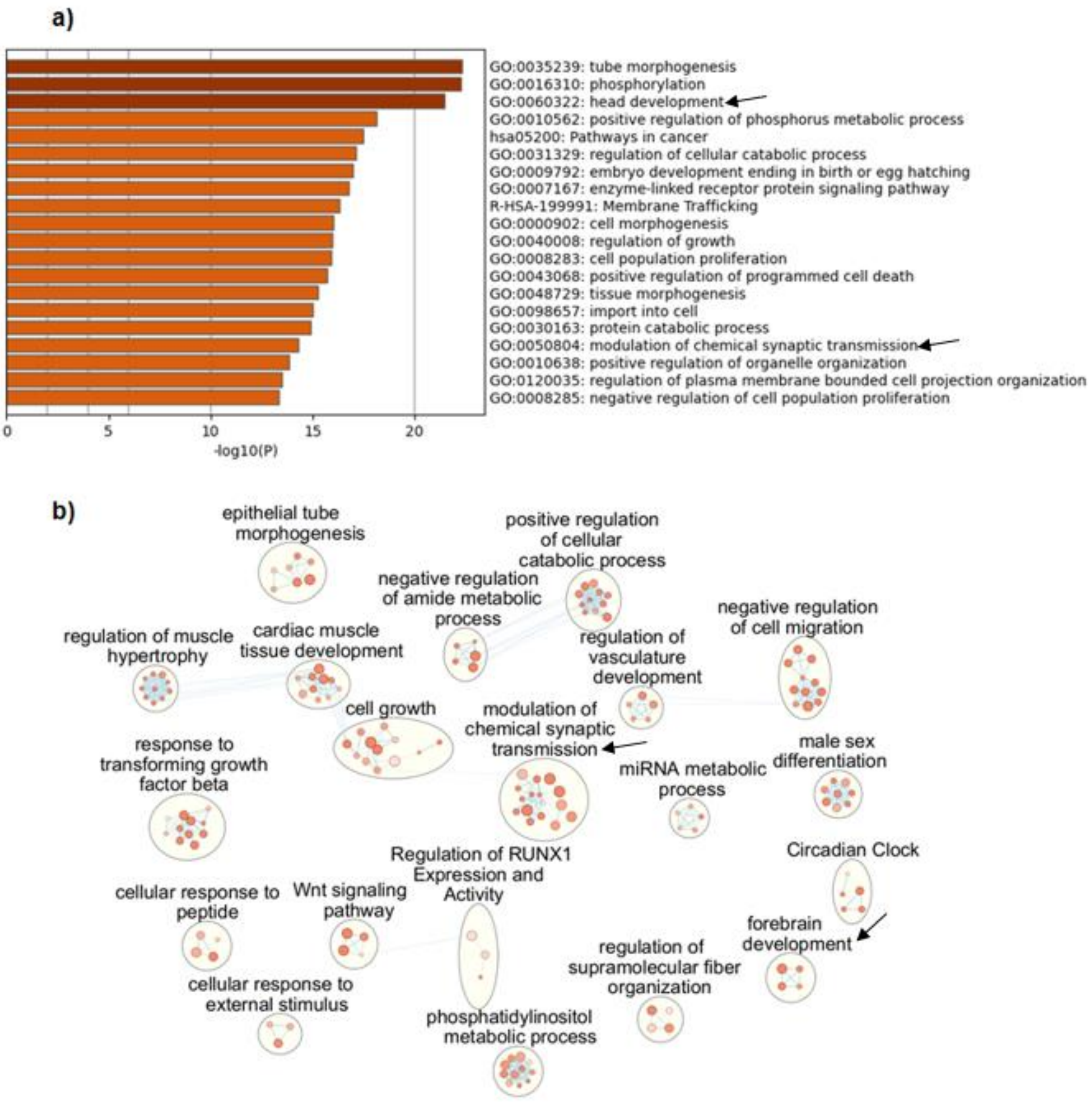

Supplementary figure 14. Pathway enrichment analysis results for genes targeted by hsa-miR-130a-3p. a) Bar graph of enriched terms found in Metascape analysis, colored by p-values; b) Clusters defined by clusterMaker2 based on gProfiler results. Arrows indicate clusters related to nervous system or neurodevelopment.

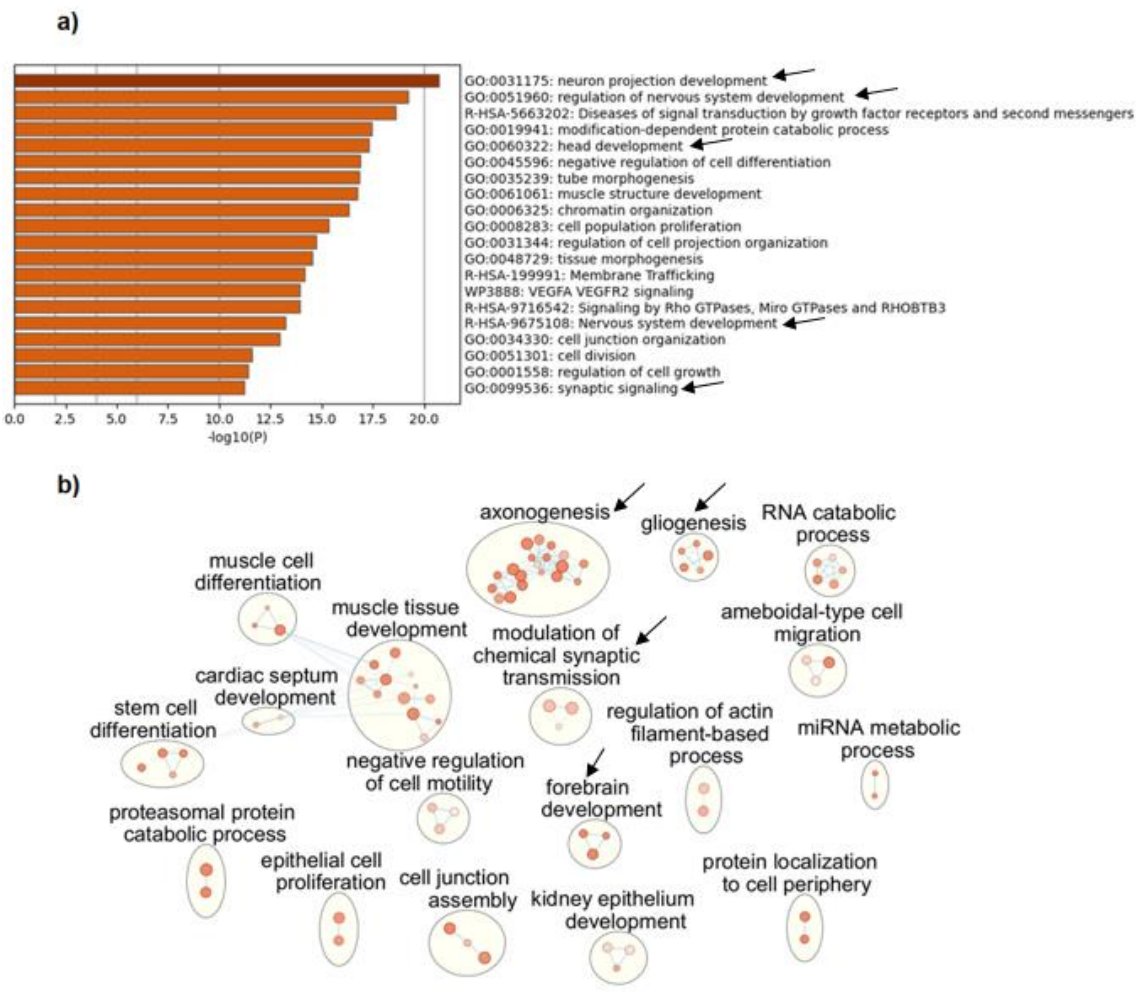

Supplementary figure 15. Pathway enrichment analysis results for genes targeted by hsa-miR-146b-5p. a) Bar graph of enriched terms found in Metascape analysis, colored by p-values; b) Clusters defined by clusterMaker2 based on gProfiler results. Arrows indicate clusters related to nervous system or neurodevelopment.

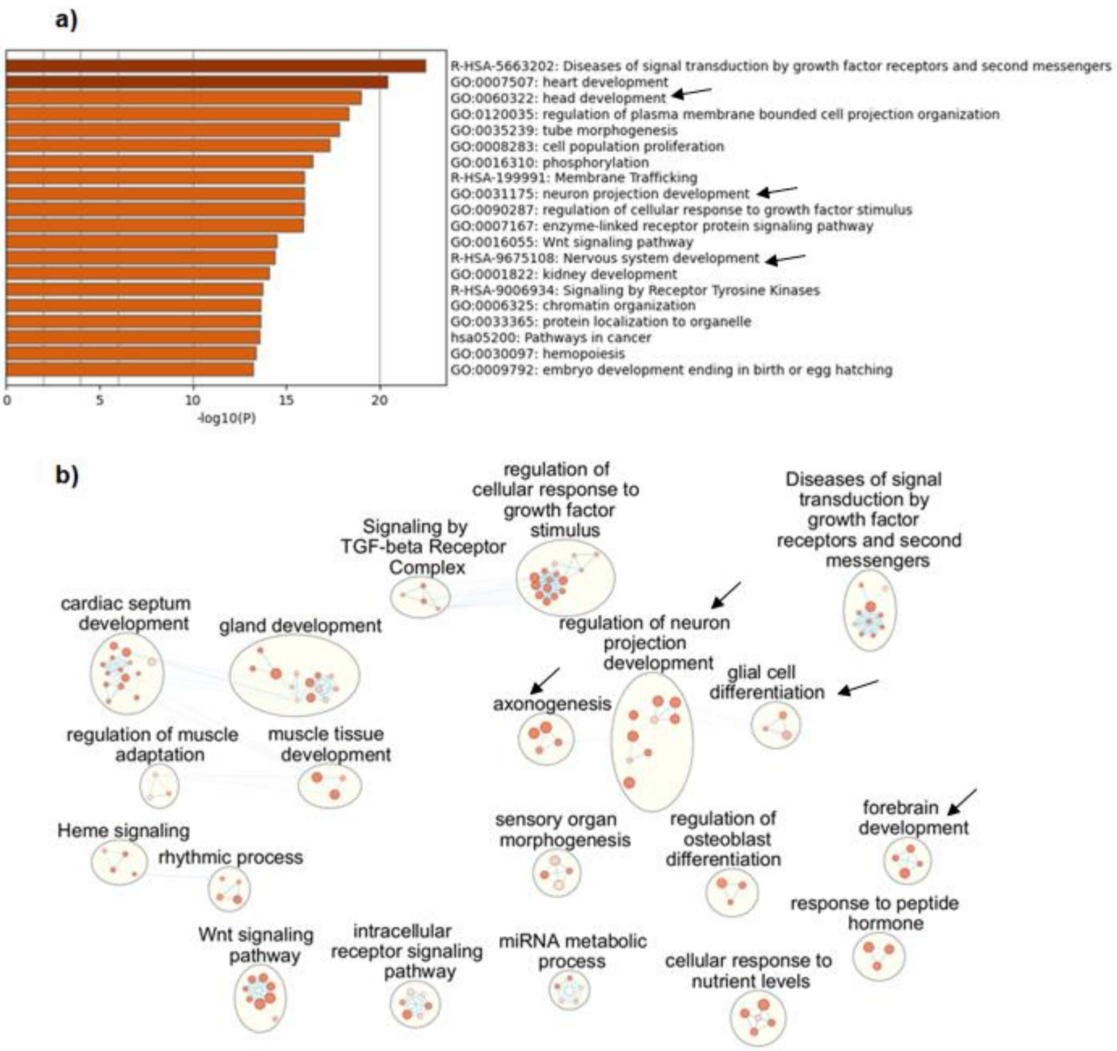

Supplementary figure 16. Pathway enrichment analysis results for genes targeted by hsa-miR-140-5p. a) Bar graph of enriched terms found in Metascape analysis, colored by p-values; b) Clusters defined by clusterMaker2 based on gProfiler results. Arrows indicate clusters related to nervous system or neurodevelopment.

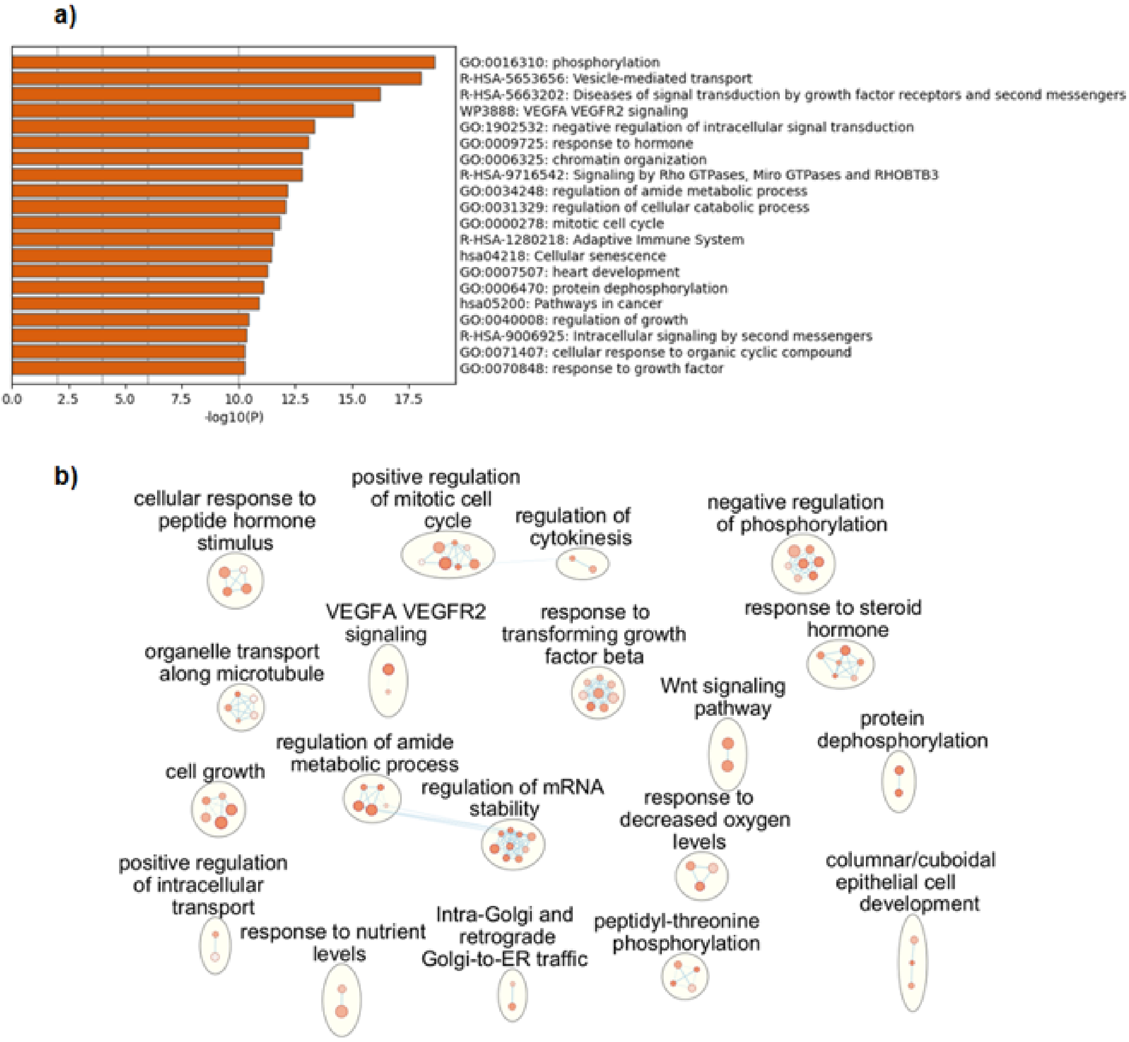

Supplementary figure 17. Pathway enrichment analysis results for genes targeted by hsa-miR-99a-5p. a) Bar graph of enriched terms found in Metascape analysis, colored by p-values; b) Clusters defined by clusterMaker2 based on gProfiler results. Arrows indicate clusters related to nervous system or neurodevelopment.

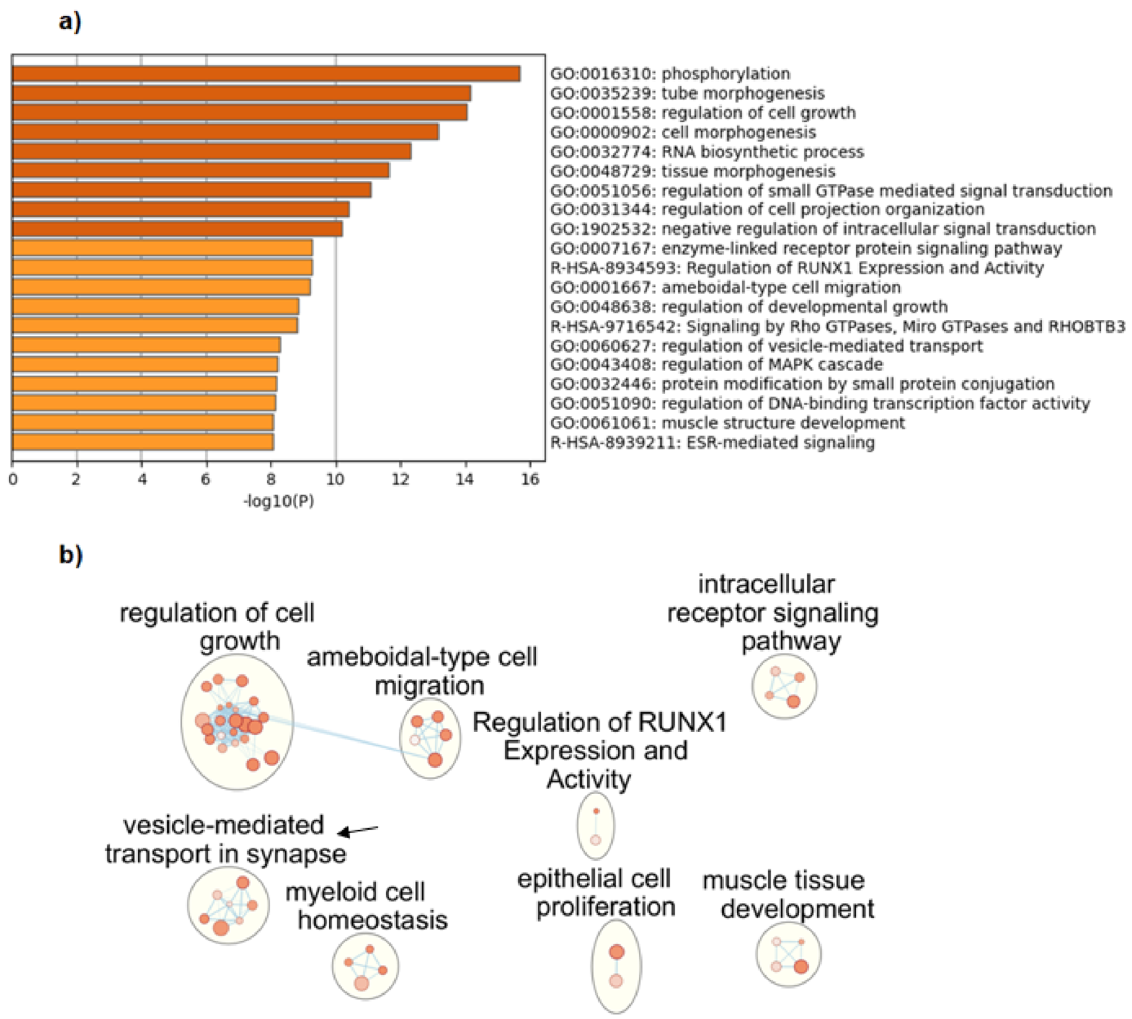

Supplementary figure 18. Pathway enrichment analysis results for genes targeted by hsa-miR-574-3p. a) Bar graph of enriched terms found in Metascape analysis, colored by p-values; b) Clusters defined by clusterMaker2 based on gProfiler results. Arrows indicate clusters related to nervous system or neurodevelopment.

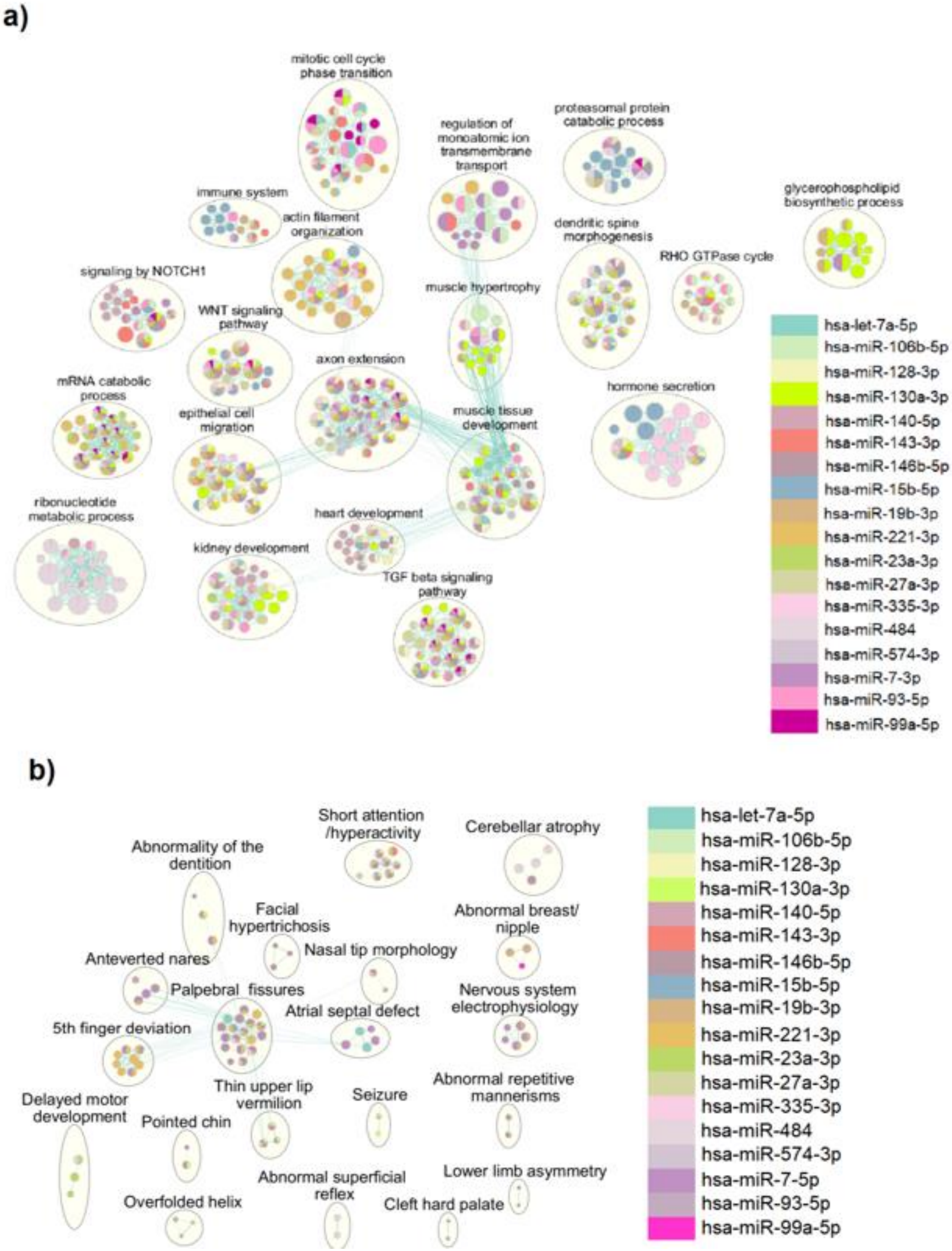

Supplementary figure 19. Enrichment map and clusters of enriched terms for miRNAs target genes. a) Enrichment map for biological pathways; b) Enrichment map for HPO terms. Colored according to miRNA target gene list.

a)

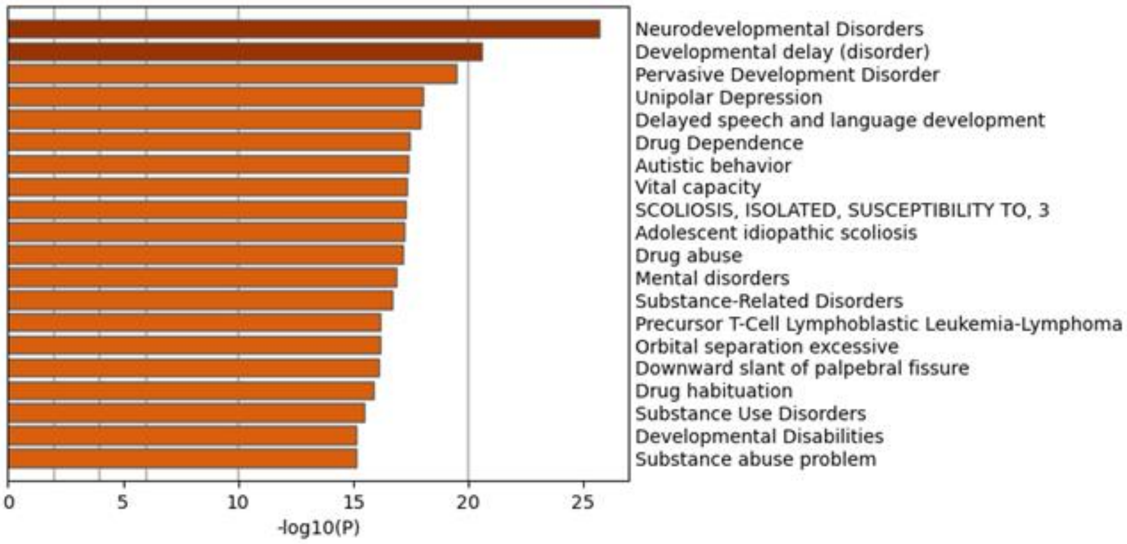

b)

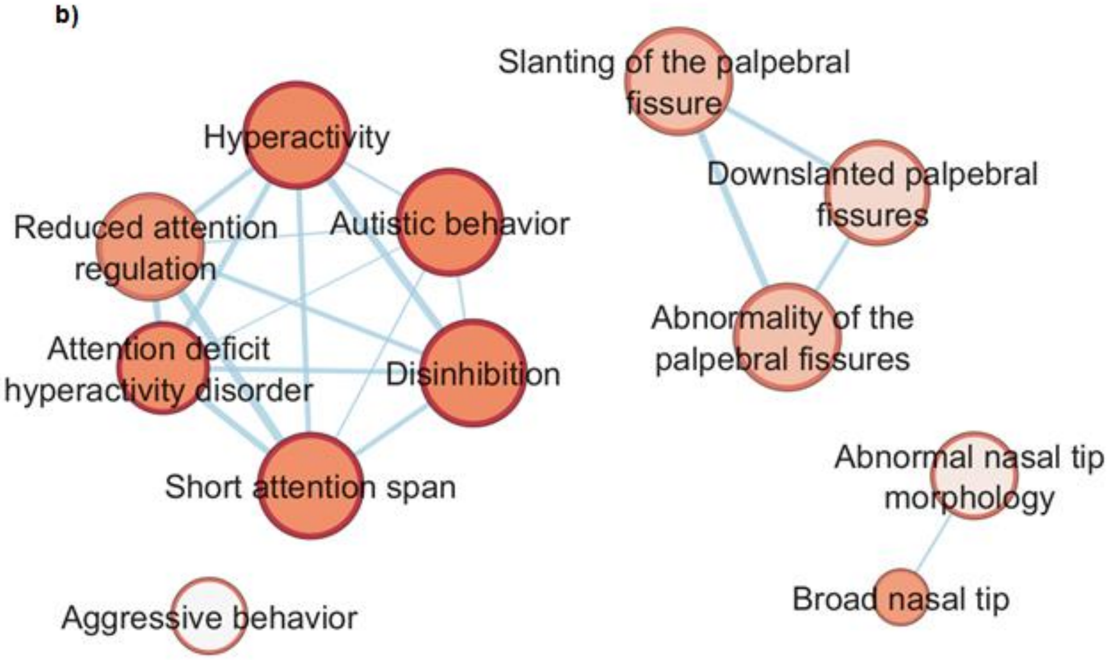

Supplementary figure 20. Enrichment analysis results in DisGeNET and HPO for genes targeted by hsa-miR-335-3p. a) Bar graph of enriched terms found in Metascape analysis, colored by p-values; b) gProfiler results for phenotypes from HPO.

a)

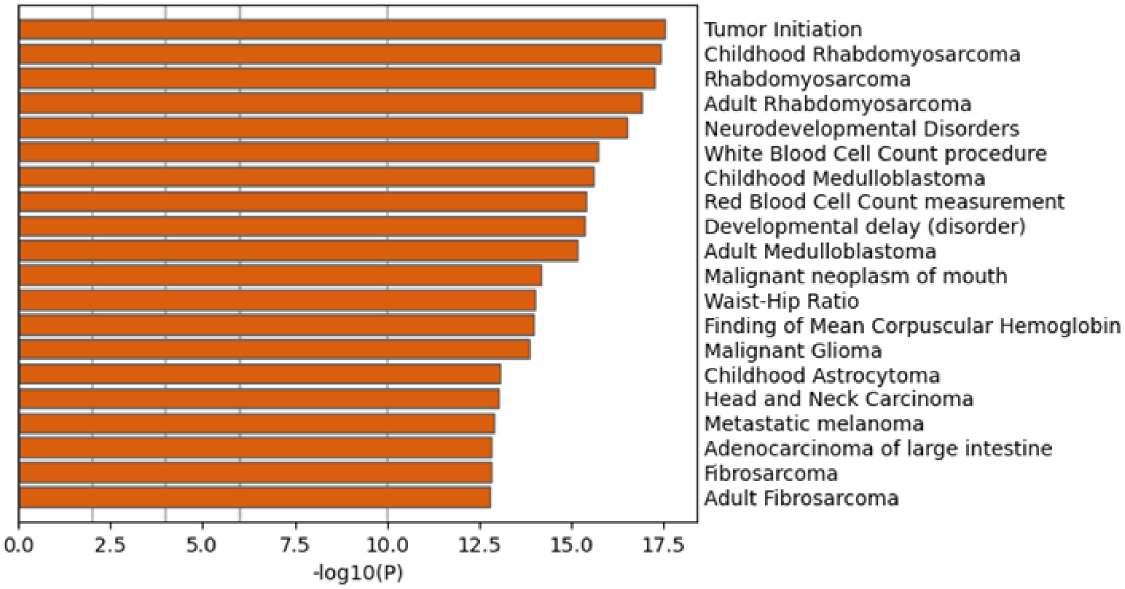

b)

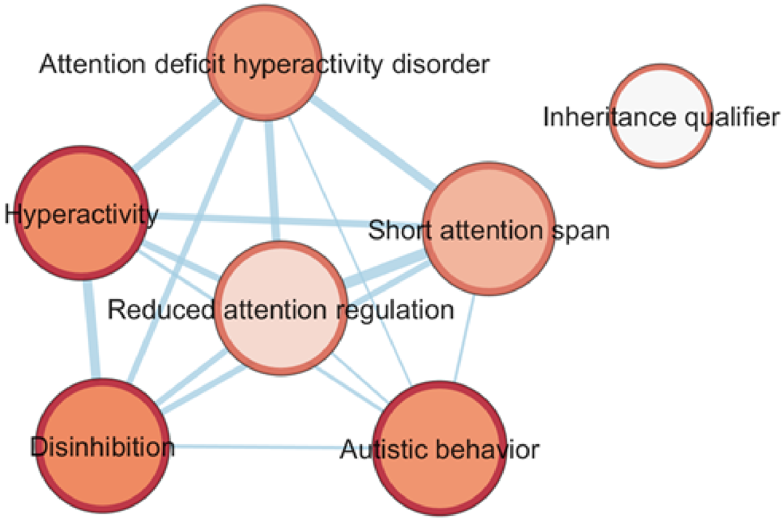

Supplementary figure 21. Enrichment analysis results in DisGeNET and HPO for genes targeted by hsa-miR-93-5p. a) Bar graph of enriched terms found in Metascape analysis, colored by p-values; b) gProfiler results for phenotypes from HPO.

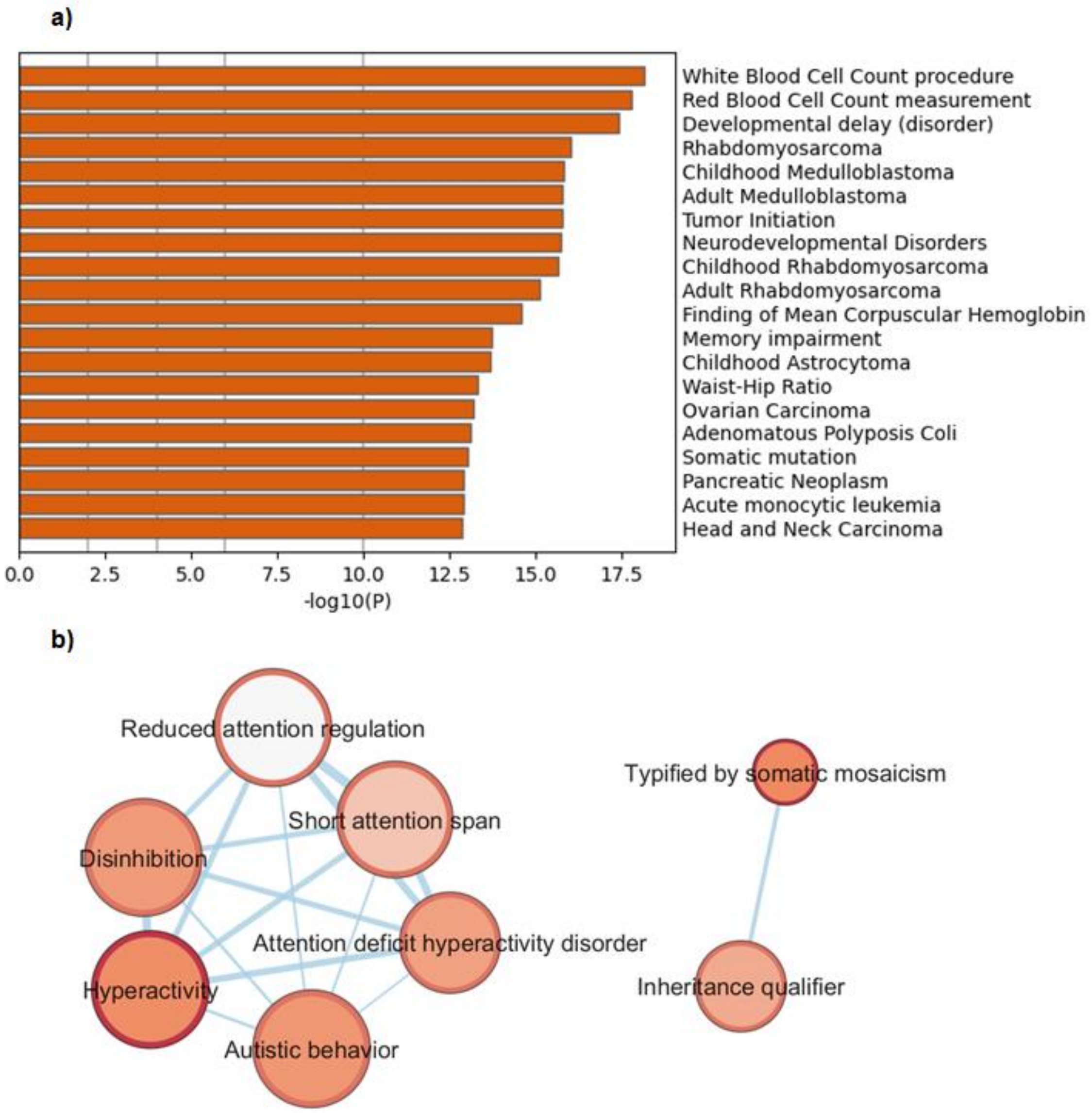

Supplementary figure 22. Enrichment analysis results in DisGeNET and HPO for genes targeted by hsa-miR-106b-5p**.** a) Bar graph of enriched terms found in Metascape analysis, colored by p-values; b) gProfiler results for phenotypes from HPO.

a)

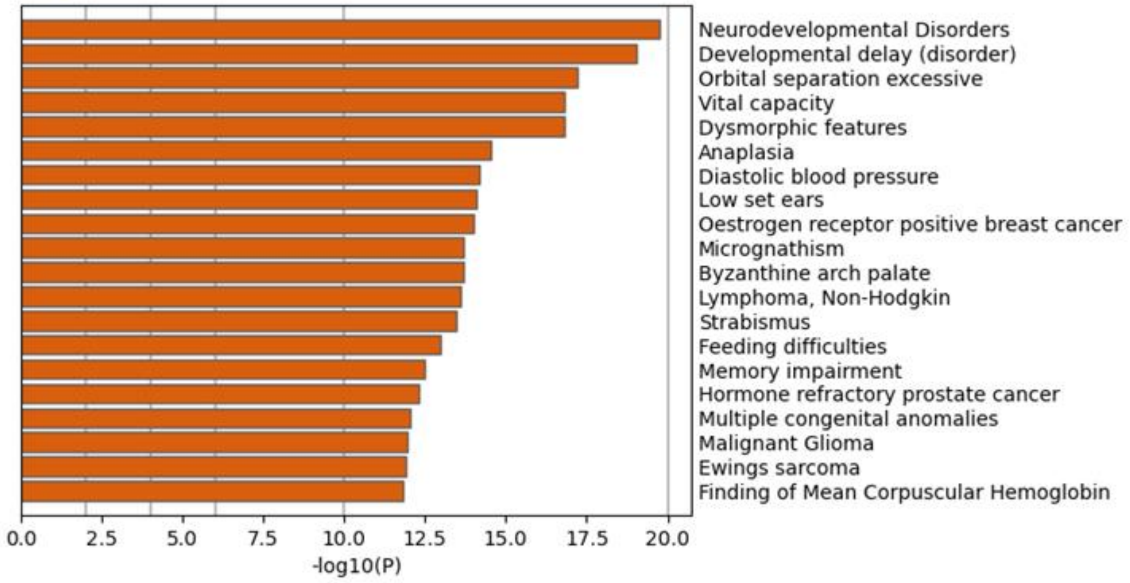

b)

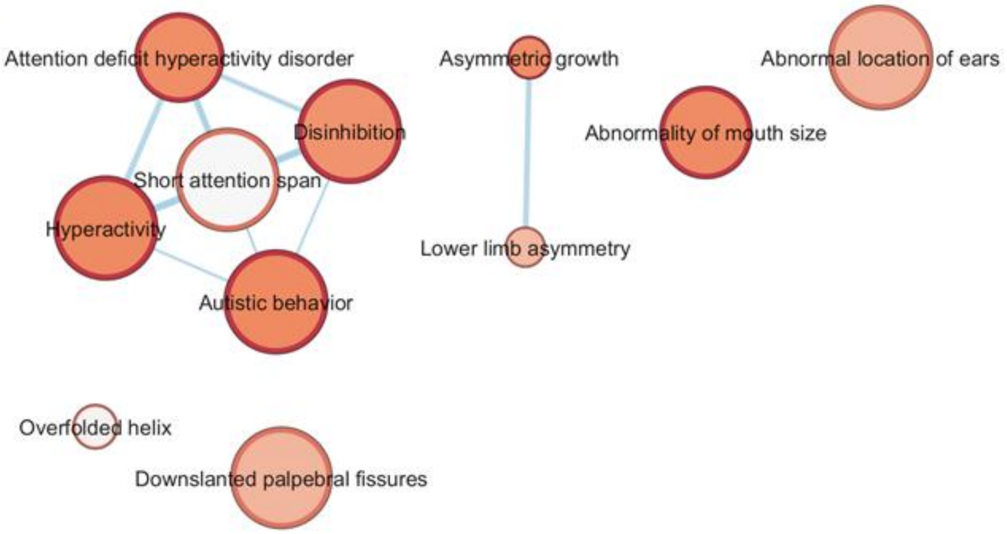

Supplementary figure 23. Enrichment analysis results in DisGeNET and HPO for genes targeted by hsa-miR-15b-5p. a) Bar graph of enriched terms found in Metascape analysis, colored by p-values; b) gProfiler results for phenotypes from HPO.

a)

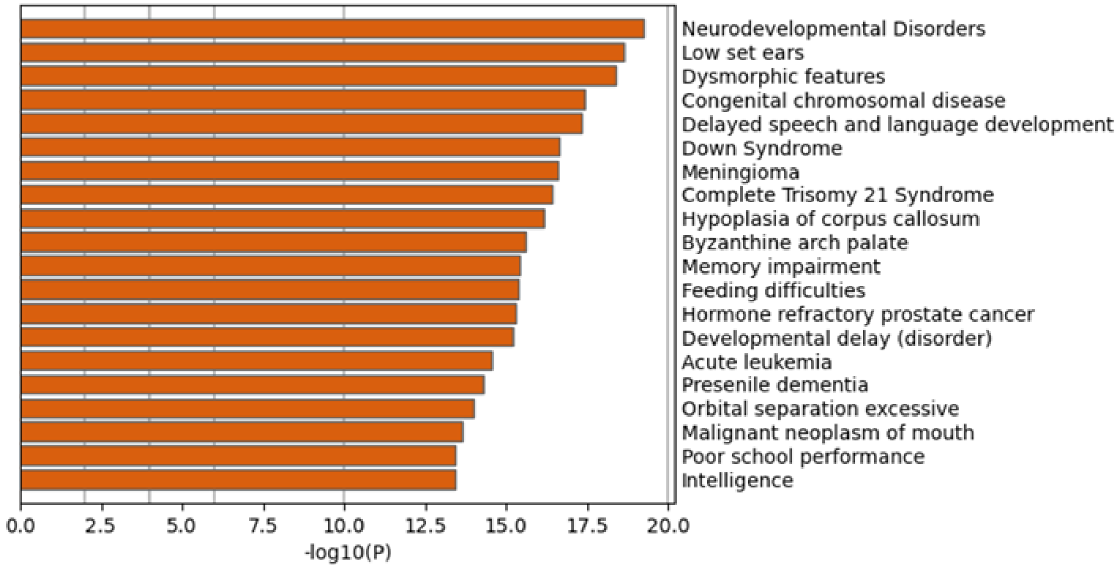

b)

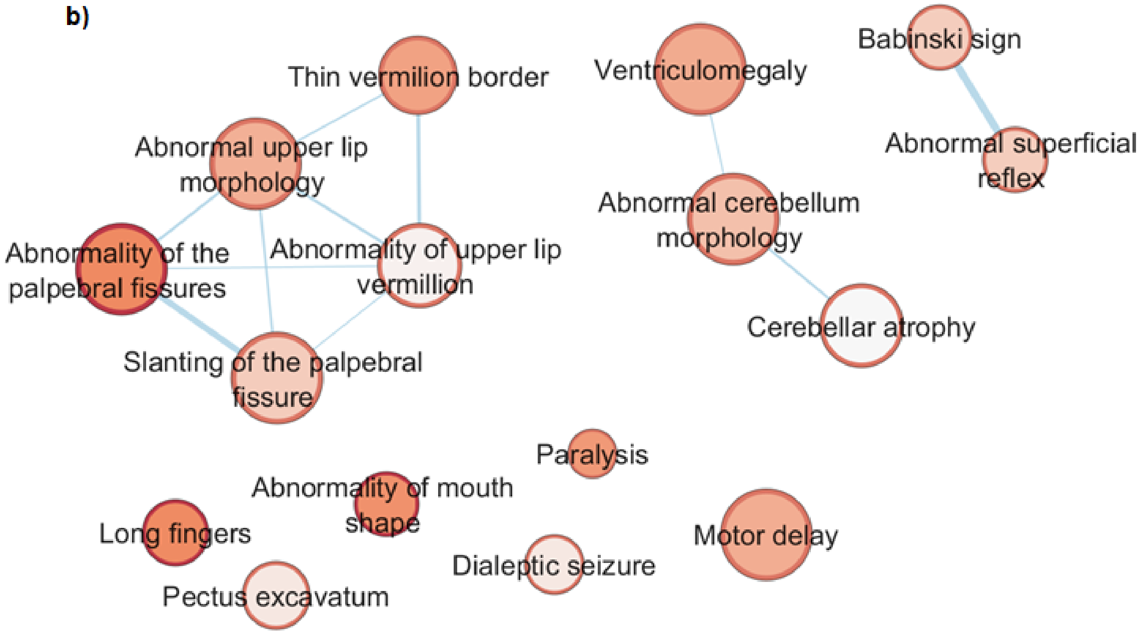

Supplementary figure 24. Enrichment analysis results in DisGeNET and HPO for genes targeted by hsa-miR-484**.** a) Bar graph of enriched terms found in Metascape analysis, colored by p-values; b) gProfiler results for phenotypes from HPO.

a)

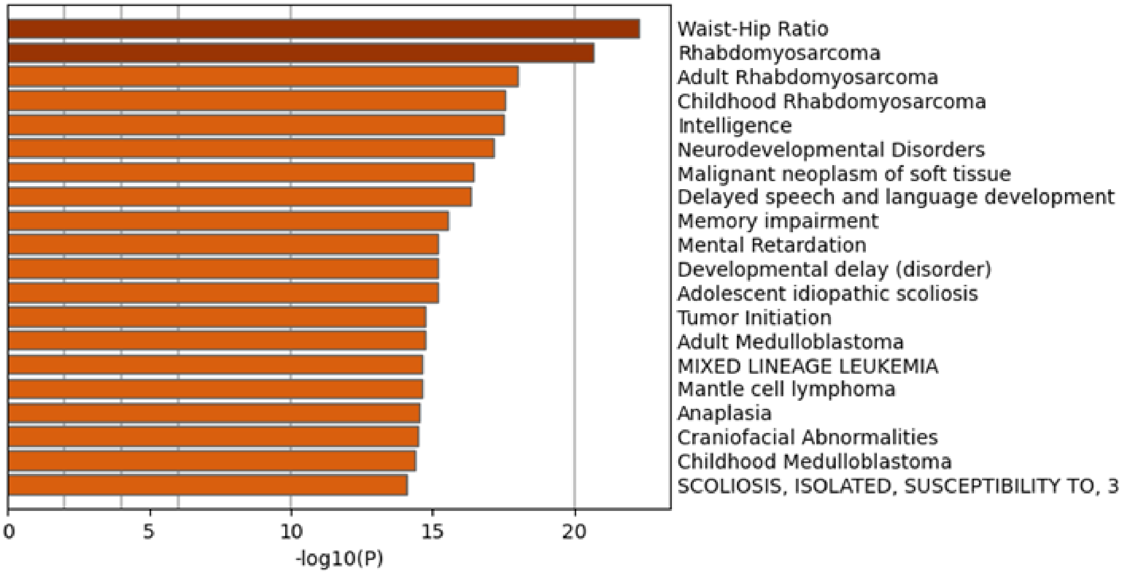

b)

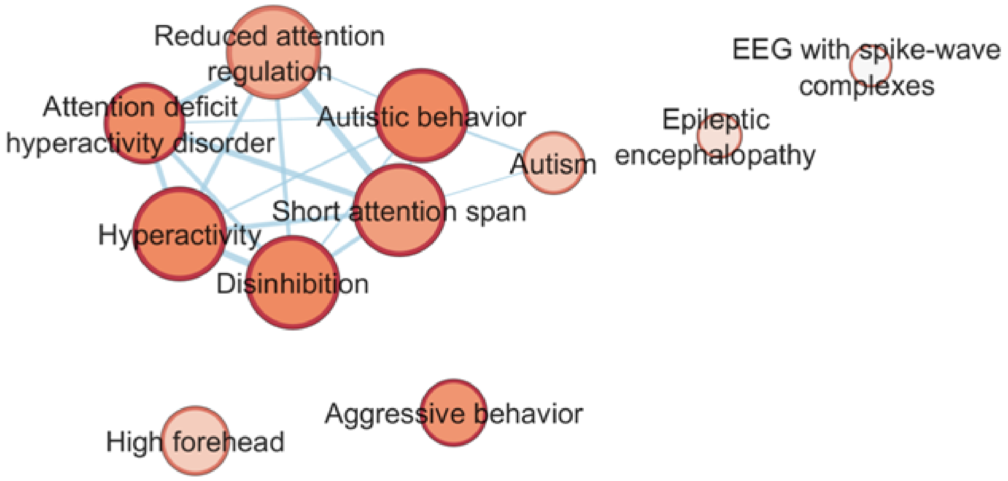

Supplementary figure 25. Enrichment analysis results in DisGeNET and HPO for genes targeted by hsa-miR-27a-3p**.** a) Bar graph of enriched terms found in Metascape analysis, colored by p-values; b) gProfiler results for phenotypes from HPO.

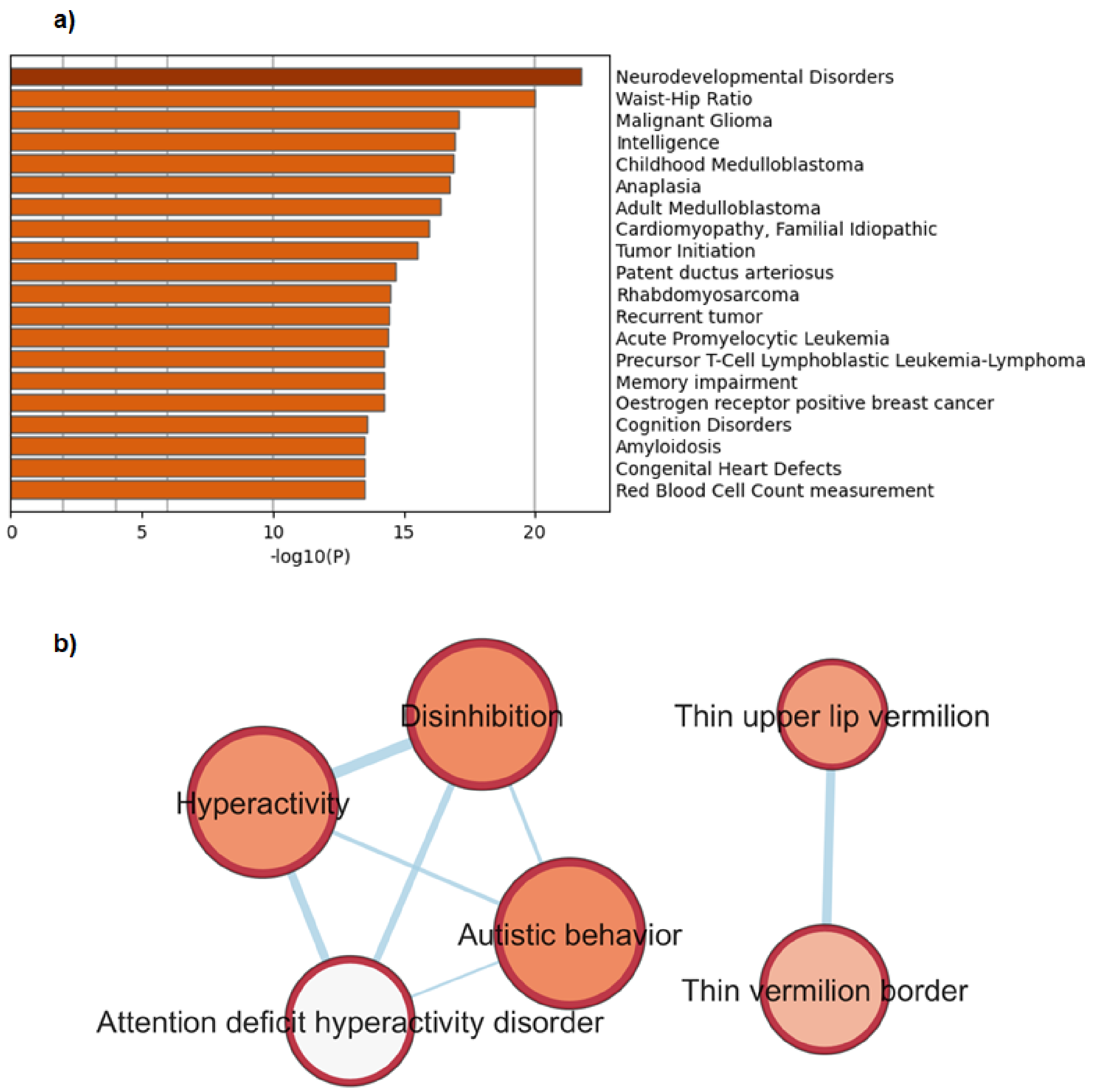

Supplementary figure 26. Enrichment analysis results in DisGeNET and HPO for genes targeted by hsa-miR-128-3p**.** a) Bar graph of enriched terms found in Metascape analysis, colored by p-values; b) gProfiler results for phenotypes from HPO.

a)

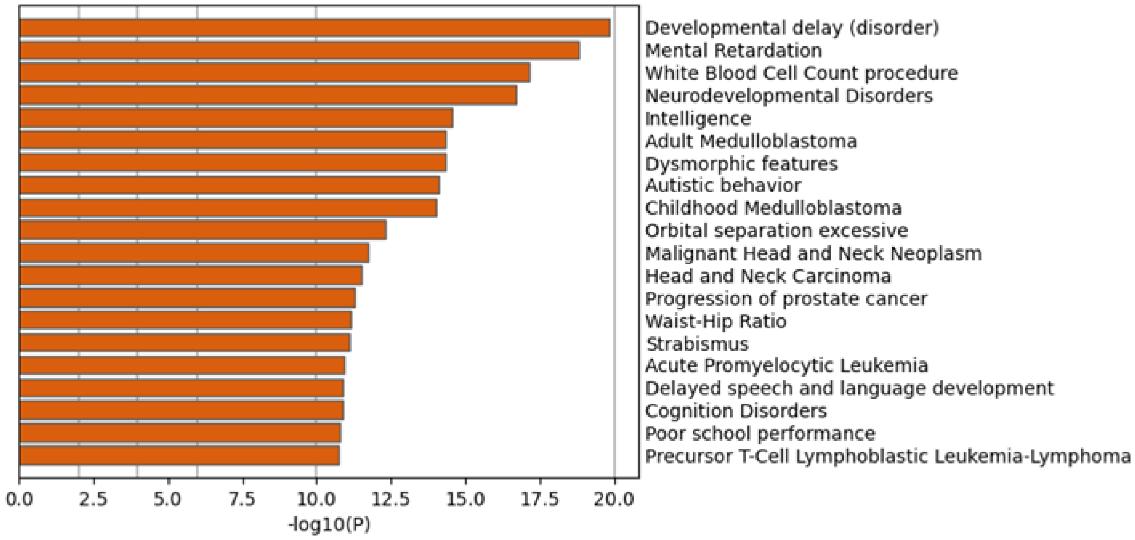

b)

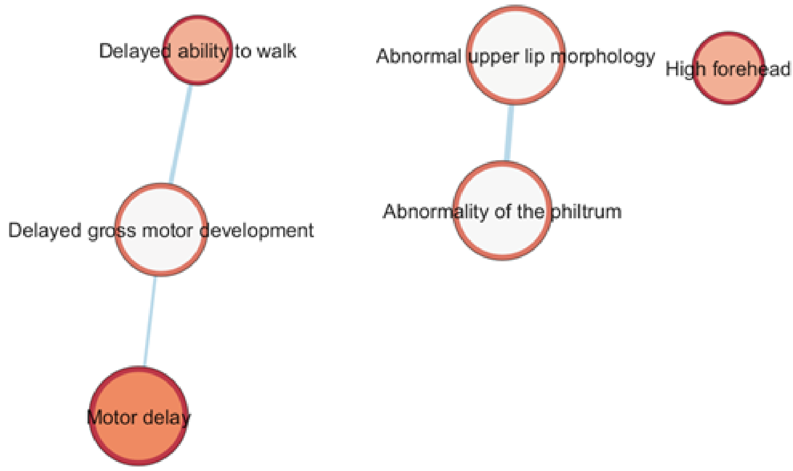

Supplementary figure 27. Enrichment analysis results in DisGeNET and HPO for genes targeted by hsa-miR-23a-3p. a) Bar graph of enriched terms found in Metascape analysis, colored by p-values; b) gProfiler results for phenotypes from HPO.

a)

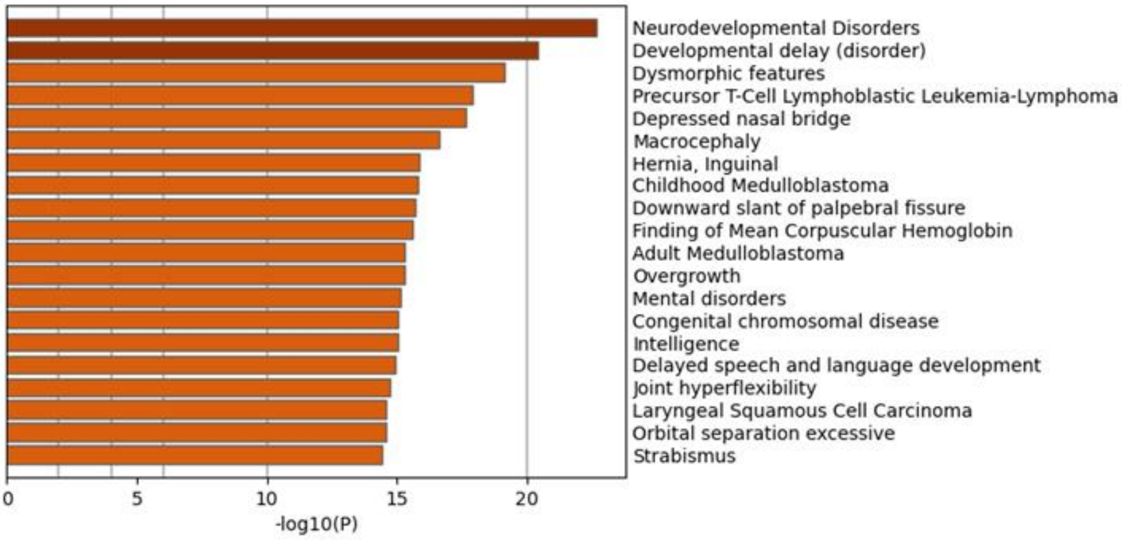

b)

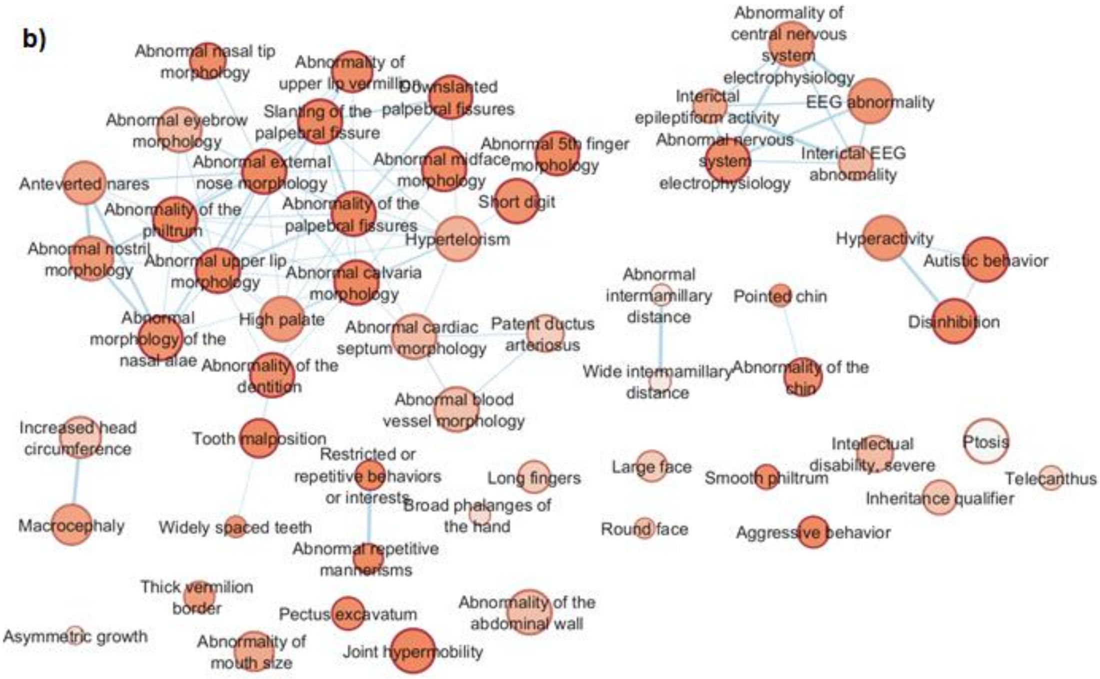

Supplementary figure 28. Enrichment analysis results in DisGeNET and HPO for genes targeted by hsa-miR-7-5p**.** a) Bar graph of enriched terms found in Metascape analysis, colored by p-values; b) gProfiler results for phenotypes from HPO.

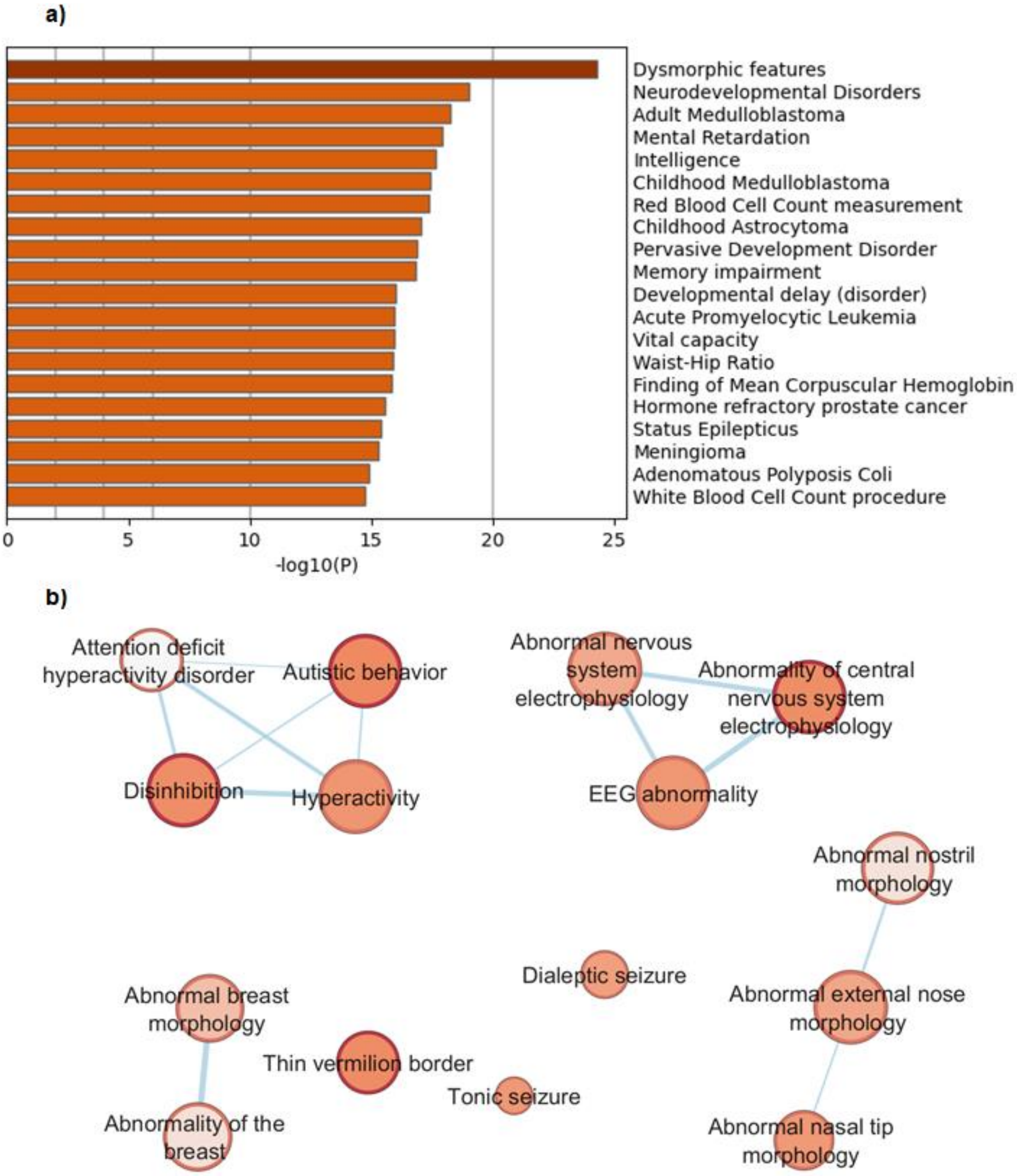

Supplementary figure 29. Enrichment analysis results in DisGeNET and HPO for genes targeted by hsa-miR-19b-3p**.** a) Bar graph of enriched terms found in Metascape analysis, colored by p-values; b) gProfiler results for phenotypes from HPO.

a)

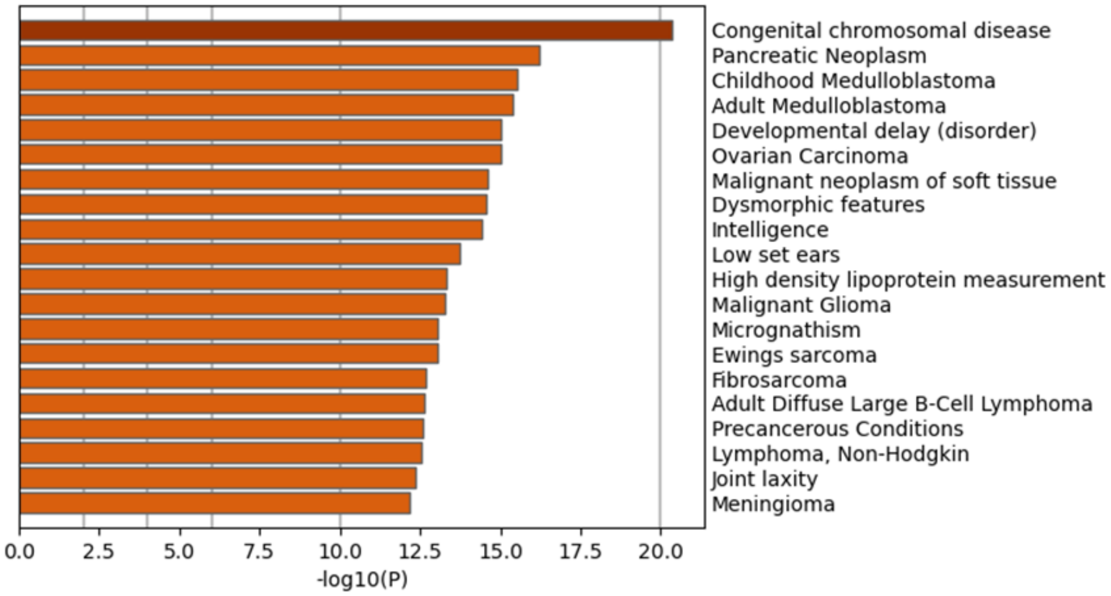

b)

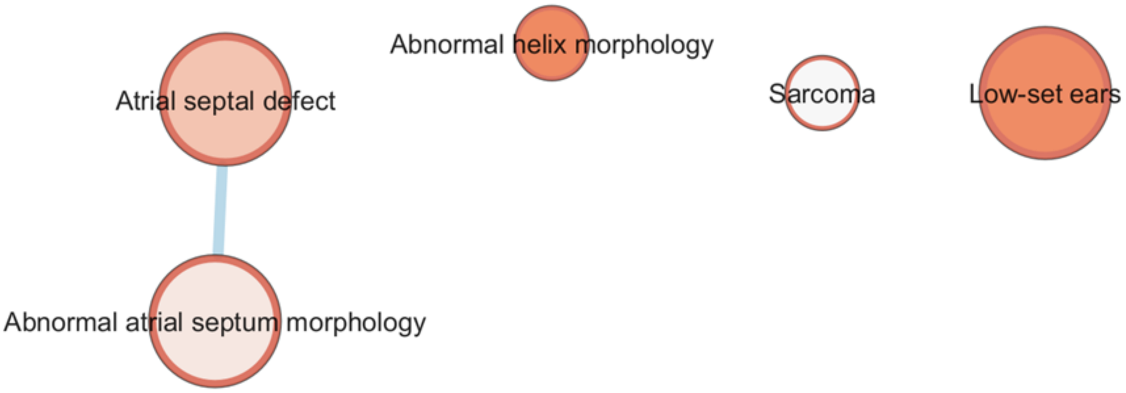

Supplementary figure 30. Enrichment analysis results in DisGeNET and HPO for genes targeted by hsa-let-7a-5p. a) Bar graph of enriched terms found in Metascape analysis, colored by p-values; b) gProfiler results for phenotypes from HPO.

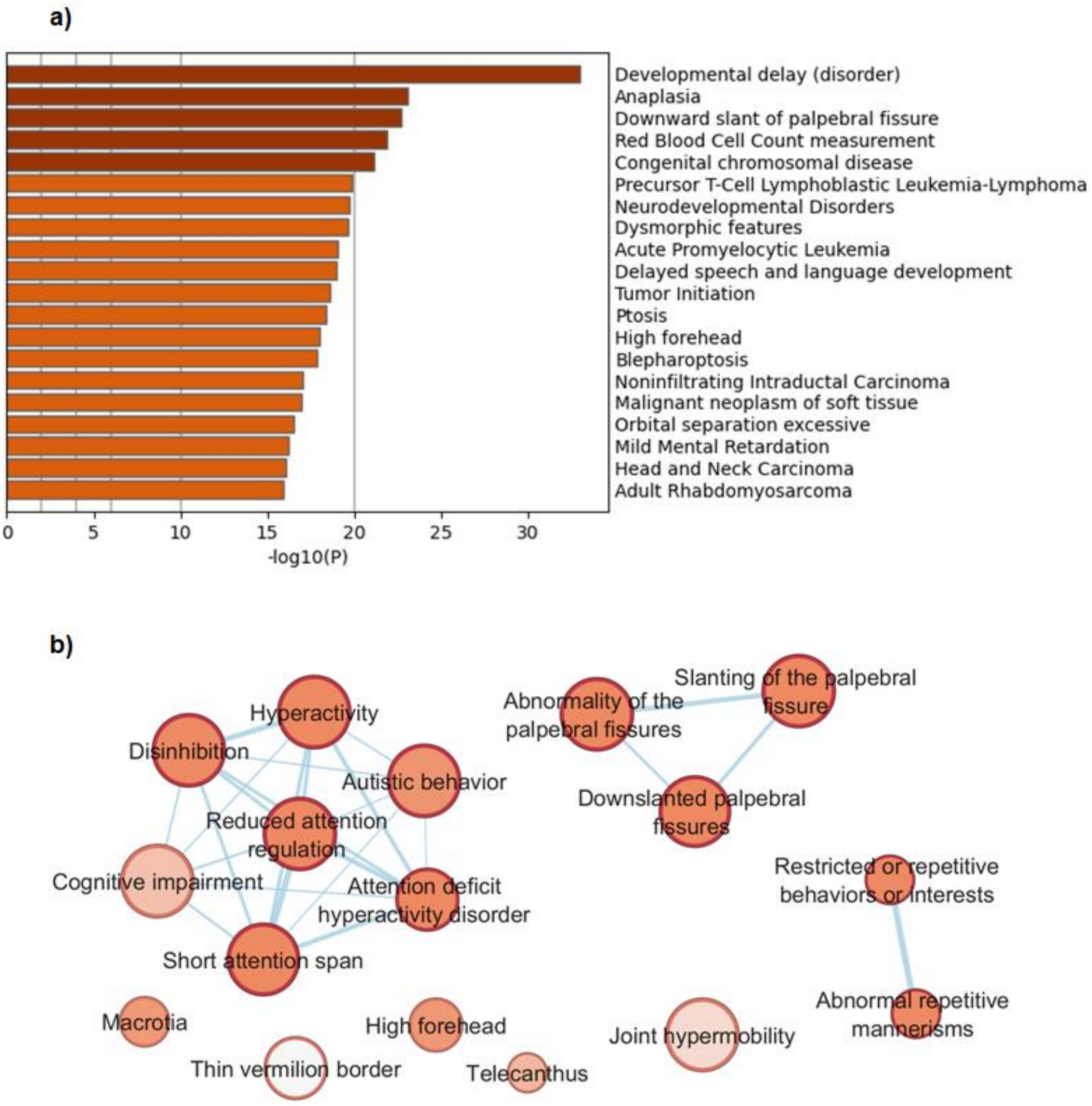

Supplementary figure 31. Enrichment analysis results in DisGeNET and HPO for genes targeted by hsa-miR-143-3p**.** a) Bar graph of enriched terms found in Metascape analysis, colored by p-values; b) gProfiler results for phenotypes from HPO.

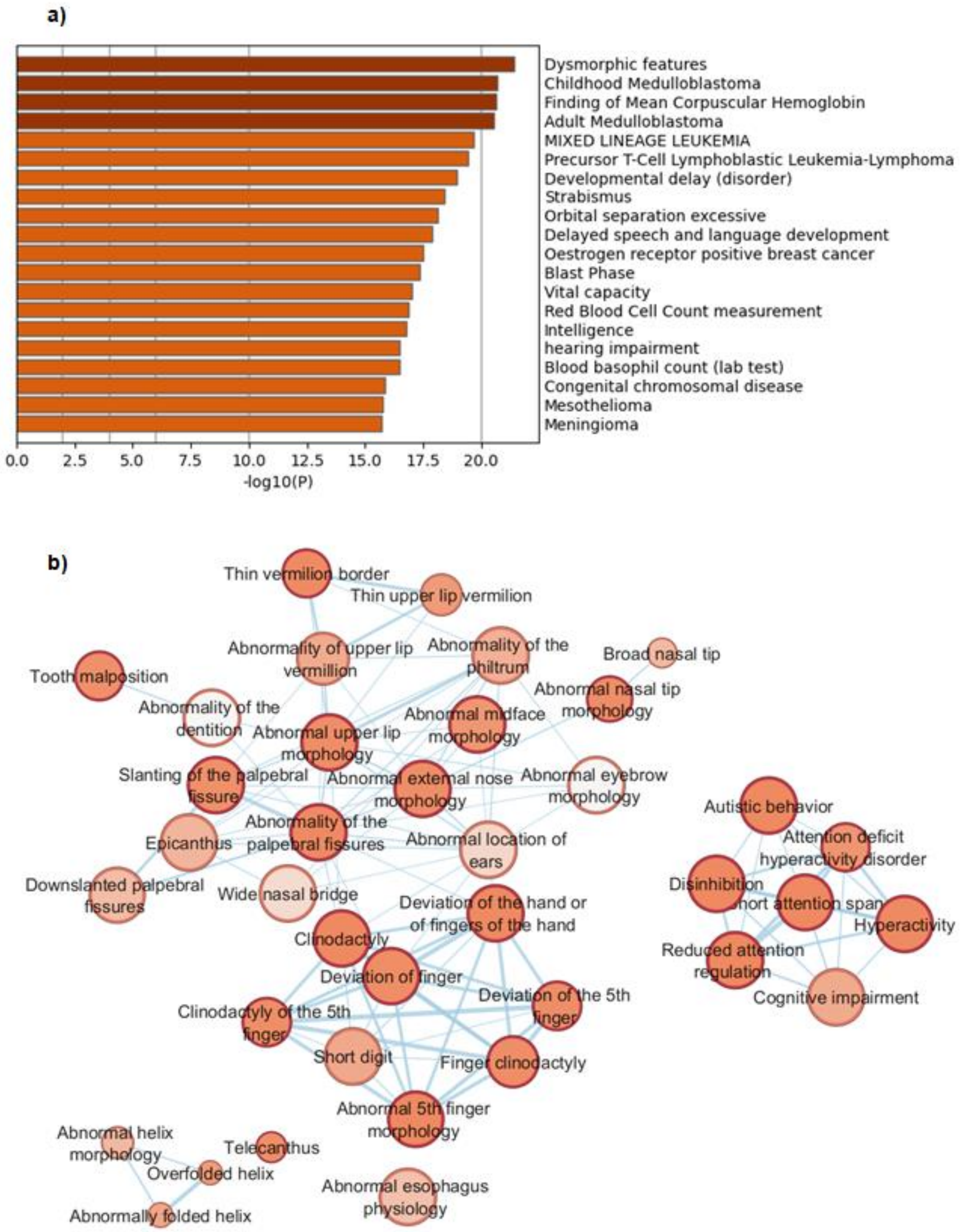

Supplementary figure 32. Enrichment analysis results in DisGeNET and HPO for genes targeted by hsa-miR-221-3p. a) Bar graph of enriched terms found in Metascape analysis, colored by p-values; b) gProfiler results for phenotypes from HPO.

a)

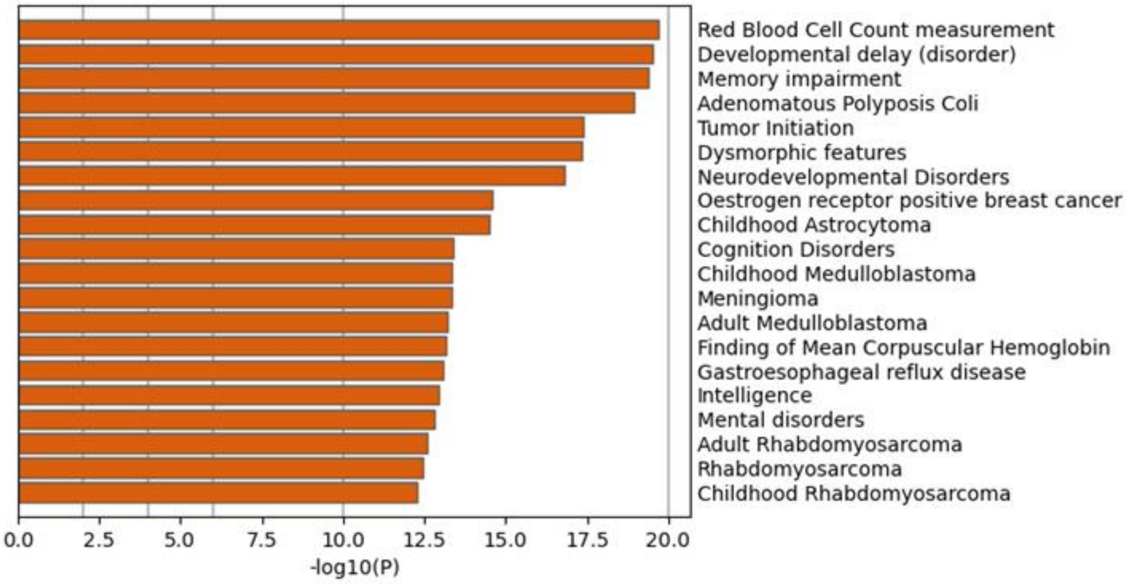

b)

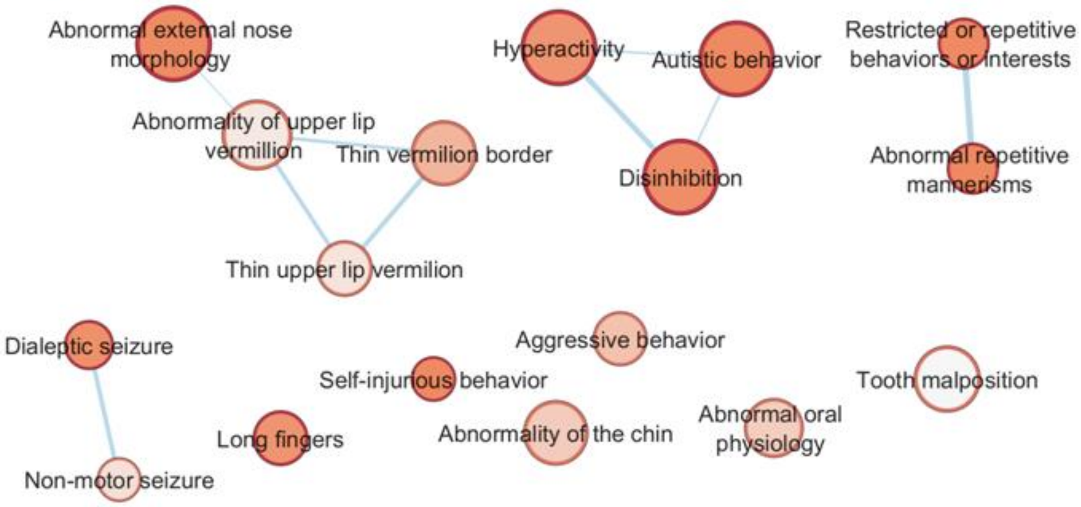

Supplementary figure 33. Enrichment analysis results in DisGeNET and HPO for genes targeted by hsa-miR-130a-3p**.** a) Bar graph of enriched terms found in Metascape analysis, colored by p-values; b) gProfiler results for phenotypes from HPO.

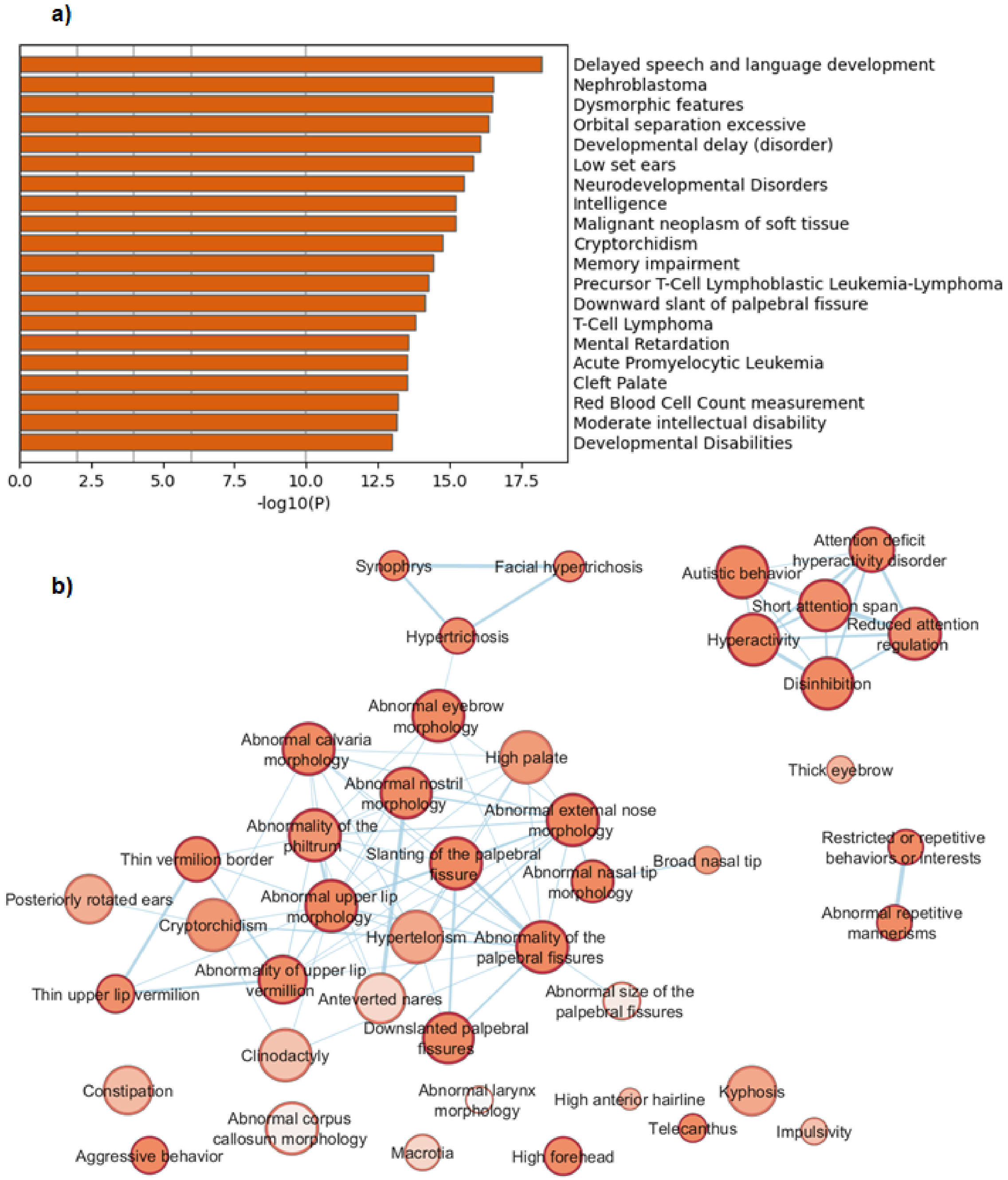

Supplementary figure 34. Enrichment analysis results in DisGeNET and HPO for genes targeted by hsa-miR-146b-5p. a) Bar graph of enriched terms found in Metascape analysis, colored by p-values; b) gProfiler results for phenotypes from HPO.

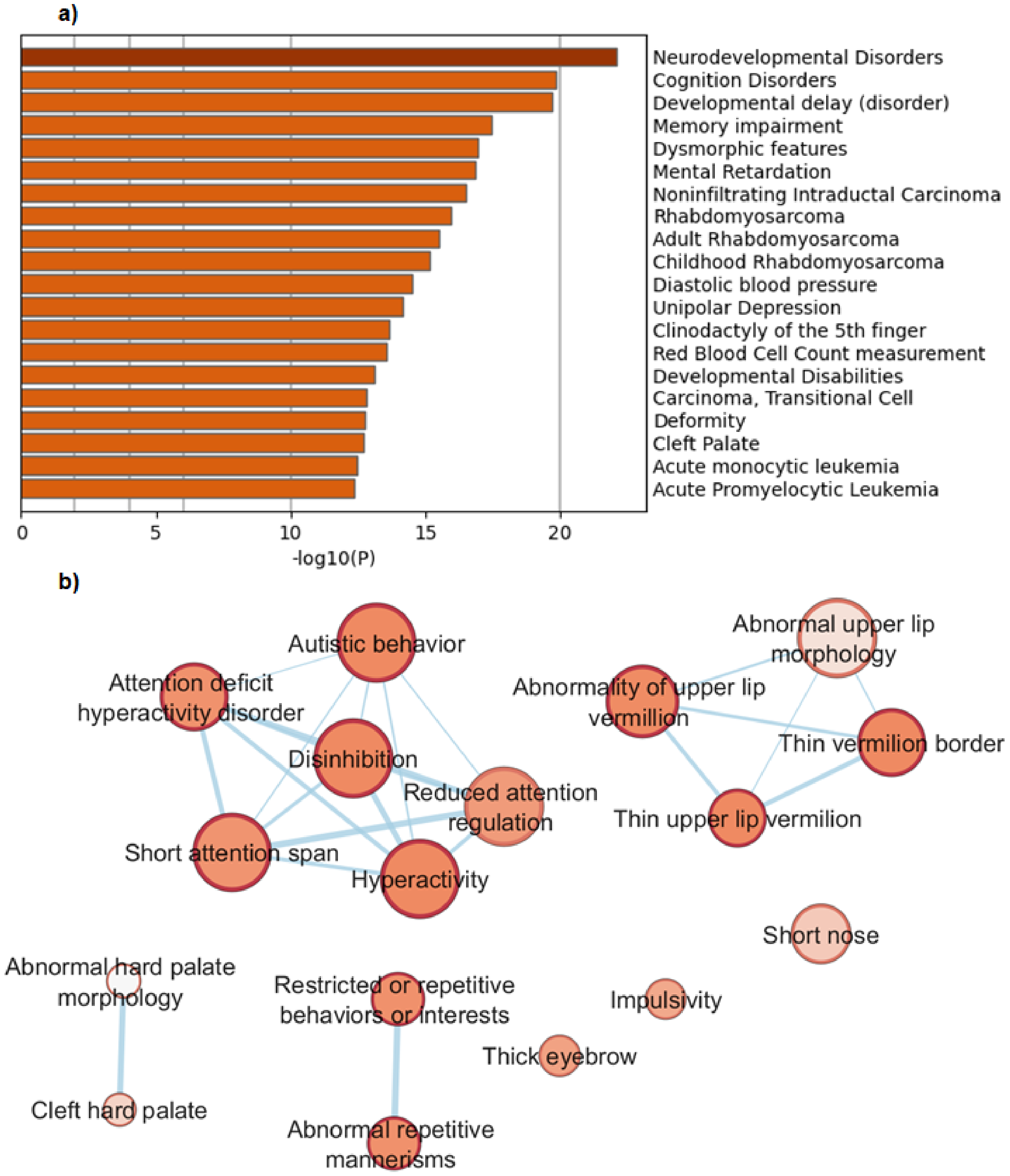

Supplementary figure 35. Enrichment analysis results in DisGeNET and HPO for genes targeted by hsa-miR-140-5p. a) Bar graph of enriched terms found in Metascape analysis, colored by p-values; b) gProfiler results for phenotypes from HPO.

a)

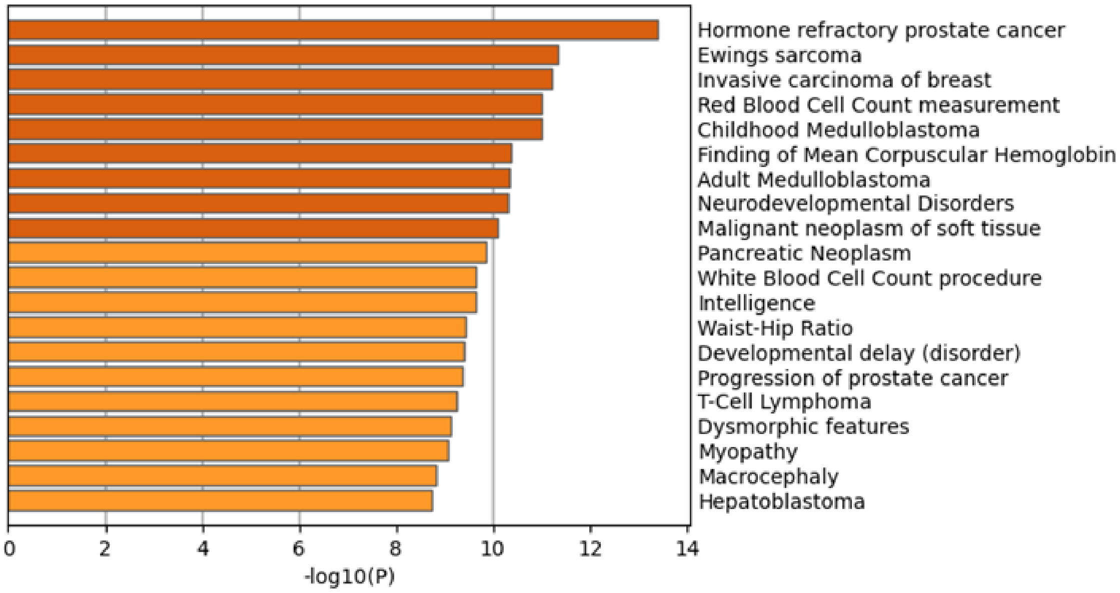

b)

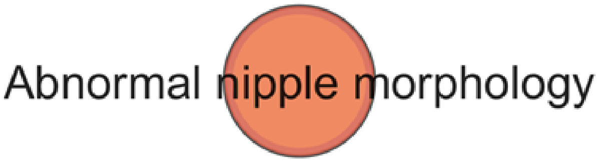

Supplementary figure 36. Enrichment analysis results in DisGeNET and HPO for genes targeted by hsa-miR-99a-5p**.** a) Bar graph of enriched terms found in Metascape analysis, colored by p-values; b) gProfiler results for phenotypes from HPO.

a)

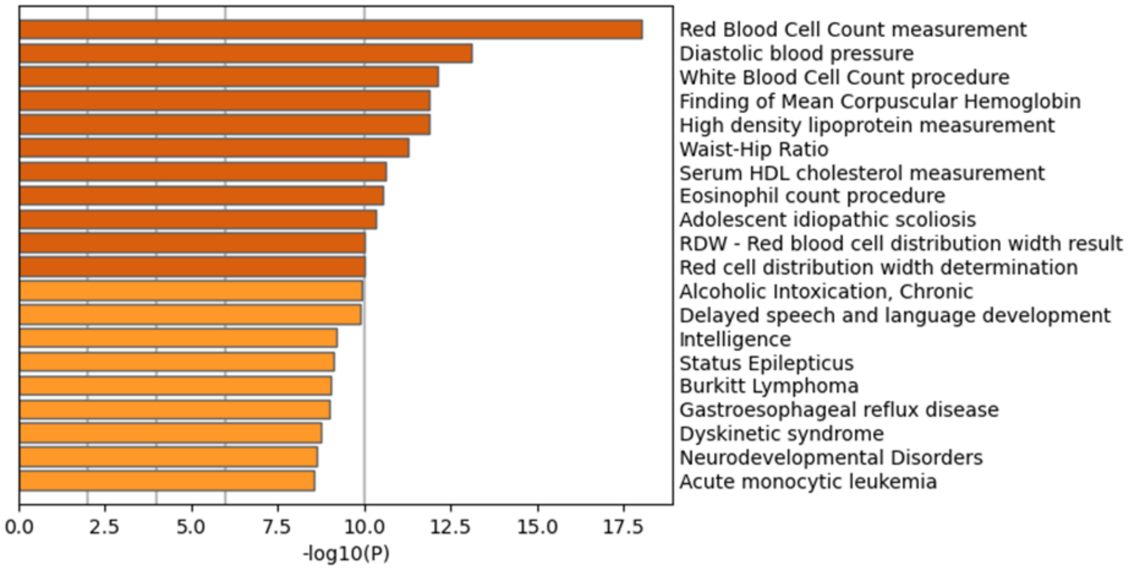

b)

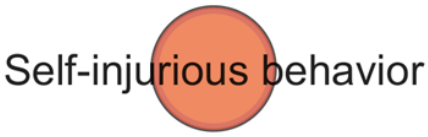

Supplementary figure 37. Enrichment analysis results in DisGeNET and HPO for genes targeted by hsa-miR-574-3p. a) Bar graph of enriched terms found in Metascape analysis, colored by p-values; b) gProfiler results for phenotypes from HPO.

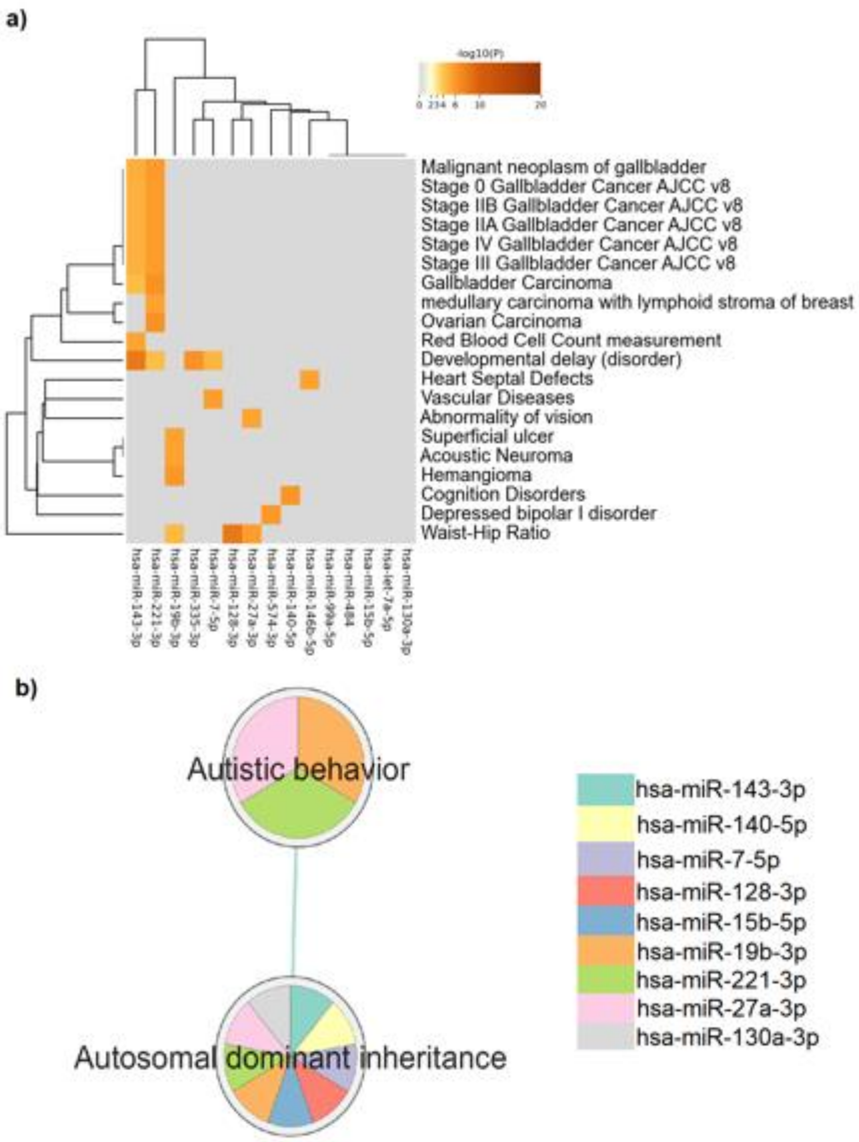

Supplementary figure 38. Enrichment analysis for diseases and phenotypes and target genes expressed in common in sperm and neurons. a) Heatmap of enriched terms from DisGeNET across miRNAs target genes list, produced by Metascape; c) Enrichment map of enriched terms from HPO for miRNAs target genes lists created using EnrichmentMap in Cytoscape and gProfiler results.

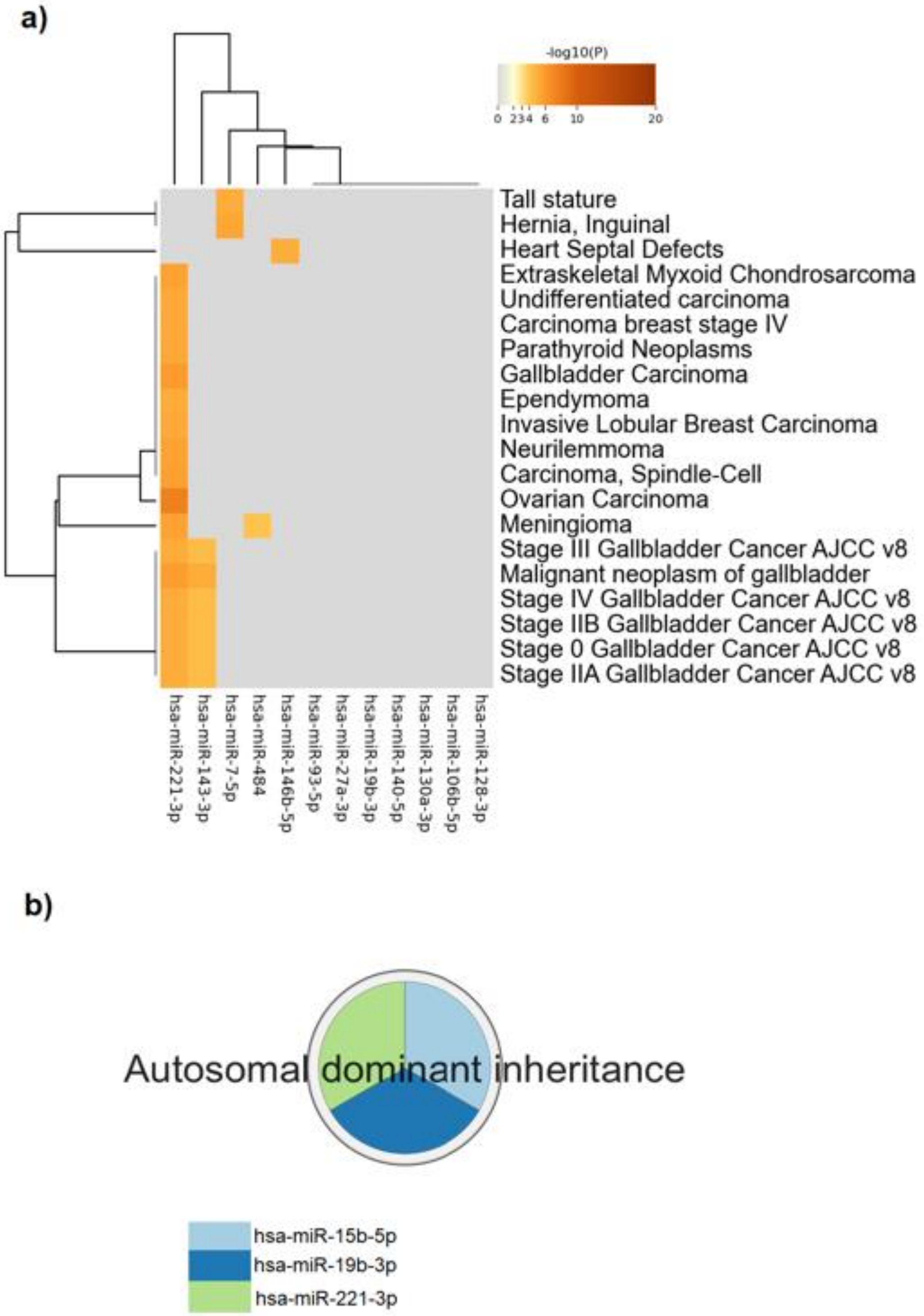

Supplementary figure 39. Enrichment analysis for diseases and phenotypes and target genes expressed in common in sperm, neurons and PBMC. a) Heatmap of enriched terms from DisGeNET across miRNAs target genes list, produced by Metascape; c) Enrichment map of enriched terms from HPO for miRNAs target genes lists created using EnrichmentMap in Cytoscape and gProfiler results.
